# Systematic enzyme and cofactor engineering for efficient ursolic acid biosynthesis in *Yarrowia lipolytica*

**DOI:** 10.1016/j.synbio.2026.05.012

**Published:** 2026-07-17

**Authors:** Hany Elsharawy, Qian He, Weizhu Zeng, Jingwen Zhou

**Affiliations:** a Science Center for Future Foods, Jiangnan University, Wuxi, 214122, China; b Department of Genetics, Faculty of Agriculture, Cairo University, Giza, Egypt; c School of Biotechnology, Jiangnan University, 1800 Lihu Road, Wuxi, Jiangsu, 214122, China; d Engineering Research Center of Ministry of Education on Food Synthetic Biotechnology, Jiangnan University, 1800 Lihu Road, Wuxi, Jiangsu, 214122, China

**Keywords:** Ursolic acid, Oe*CYP716A48*, Cofactor engineering, Enzyme engineering, *Yarrowia lipolytica*

## Abstract

Ursolic acid (UA) is a pharmaceutically valuable pentacyclic triterpenoid, but its microbial production is constrained by inefficient cytochrome P450 catalysis and limited cofactor availability. Here, we engineered an efficient α-amyrin–producing *Yarrowia lipolytica* chassis for UA biosynthesis through integrated enzyme engineering, cofactor optimization, and metabolic flux balancing. Screening of heterologous plant cytochrome P450 monooxygenases identified *CYP716A48* from *Olea europaea* as the most efficient α-amyrin oxidase in *Y. lipolytica*. Fusion of Oe*CYP716A48* with its redox partner *AtCPR1* using optimized flexible linkers enhanced intramolecular electron transfer and significantly increased UA titers. The Oe*CYP716A48*^D114Q/L211F^ variant enlarged the substrate-access tunnel and improved catalytic efficiency, resulting in a 5-fold increase in UA production. To support high Oe*CYP716A48* activity, intracellular FAD, heme, and iron availability were systematically enhanced, leading to a 14.4-fold increase in UA production. Multicopy integration of *CrMAS*, *OeCYP716A48*^D114Q/L211F^, and *AtCPR1* at rDNA loci further balanced pathway flux. In 5-L fed-batch fermentation, the engineered strain produced 813 ± 24 mg/L UA, representing the highest titer reported in *Y. lipolytica* to date. This study establishes a scalable and broadly applicable engineering strategy to overcome cytochrome P450 limitations and enable efficient triterpenoid biosynthesis in yeast.

## 1 Introduction

Ursolic acid (UA) is a naturally occurring pentacyclic triterpenoid widely distributed in medicinal plants and has attracted considerable attention due to its broad pharmacological activities, including anti-inflammatory, antioxidant, antimicrobial, anticancer, antidiabetic, and hepatoprotective effects [1,2]. Owing to these bioactivities, UA is regarded as a high-value compound with significant pharmaceutical and nutraceutical potential [3,4]. In addition, UA serves as an important biosynthetic precursor and structural scaffold for the production of other high-value triterpenoids, further enhancing its industrial relevance [5,6]. UA is predominantly obtained through plant extraction; however, this approach suffers from low yields, high energy consumption, seasonal variability, and limited scalability, rendering it insufficient to meet growing industrial demand [7,8]. Consequently, the development of sustainable and efficient alternative production strategies is urgently required. Recent advances in metabolic engineering and synthetic biology have established microbial cell factories as promising platforms for triterpenoid biosynthesis [9,10].

**Fig. 1:**
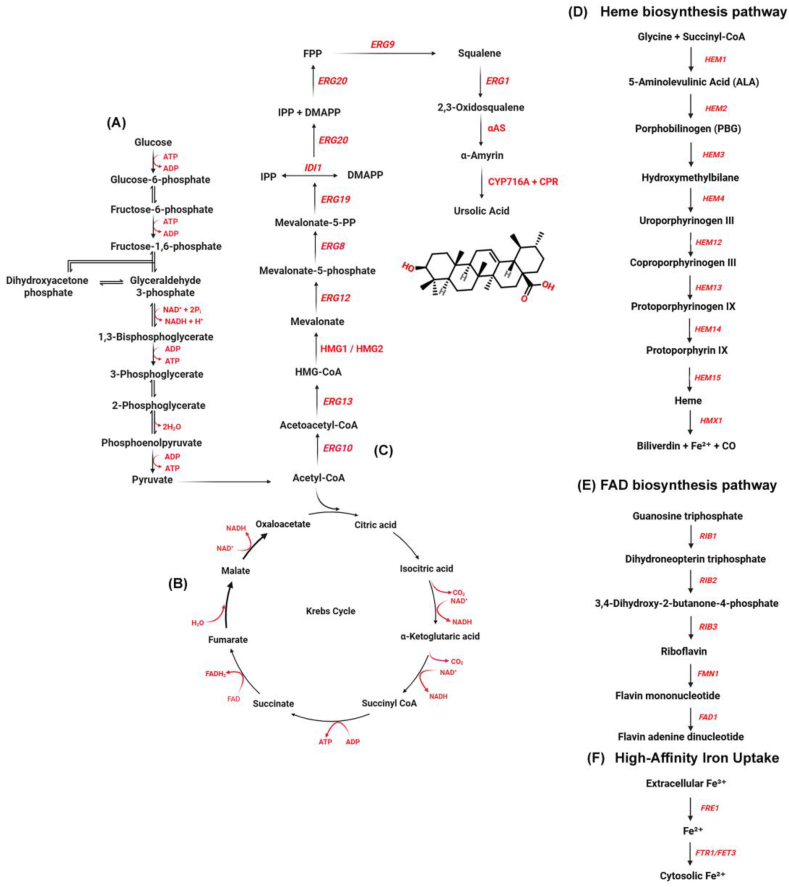
Systematic metabolic engineering strategy for enhanced ursolic acid biosynthesis in *Y. lipolytica*. (A) Glycolysis pathway supplying pyruvate and acetyl-CoA from glucose, providing the primary carbon flux for the mevalonate pathway. (B) Tricarboxylic acid (TCA) cycle generating NADH, FADH_2_, ATP, and succinyl-CoA, supporting cellular energy balance and providing precursors for heme biosynthesis. (C) Mevalonate pathway and triterpenoid biosynthetic route leading to ursolic acid production. Acetyl-CoA is converted to HMG-CoA (*ERG10*, *ERG13*) and reduced to mevalonate (*HMG1*/*HMG2*), followed by sequential phosphorylation and decarboxylation steps (*ERG12*, *ERG8*, *ERG19*, *IDI1*, *ERG20*) to form FPP, which is converted to squalene (*ERG9*), 2,3-oxidosqualene (*ERG1*), and α-amyrin (*αAS*), and finally oxidized to ursolic acid by CYP716A-CPR. (D) Heme biosynthesis pathway providing the prosthetic group required for cytochrome P450 activity. Glycine and succinyl-CoA are converted to 5-aminolevulinic acid by *HEM1*, followed by sequential reactions catalyzed by *HEM2*, *HEM3, HEM4, HEM12, HEM13, HEM14*, and *HEM15* to produce heme. Heme degradation by *HMX1* releases iron, linking heme metabolism with cellular iron homeostasis. (E) FAD biosynthesis pathway supplying flavin cofactors required for cytochrome P450 reductase activity. Guanosine triphosphate is converted to riboflavin via *RIB1, RIB2, and RIB3*, followed by phosphorylation (*FMN1*) and adenylation (*FAD1*) to generate FAD. (F) High-affinity iron uptake system ensuring sufficient intracellular Fe^2+^ for heme formation and P450 function. Extracellular Fe^3+^ is reduced by *FRE1* and transported into the cytosol via the *FTR1/FET3* complex. Red labels indicate genes targeted or pathway reinforcement to improve cofactor availability, electron transfer efficiency, and catalytic activity, thereby enhancing ursolic acid production.

The UA biosynthetic pathway begins with the conversion of squalene to 2,3-oxidosqualene by squalene epoxidase (SQE), followed by cyclization of 2,3-oxidosqualene to α-amyrin by oxidosqualene cyclase (OSC). The final step involves C-28 oxidation of α-amyrin to UA, catalyzed by a plant cytochrome P450 monooxygenase of the CYP716A family in cooperation with cytochrome P450 reductase (CPR) (Fig. 1C). Several studies have demonstrated UA production in engineered yeasts [4,6,7,11]. A modular metabolic engineering approach enhanced UA production in *Saccharomyces cerevisiae*, resulting in a titer of 8.59 g/L [12]. Although *S. cerevisiae* currently achieves higher UA titers, *Y. lipolytica* has emerged as a particularly attractive chassis for triterpenoid production due to its oleaginous nature, high intracellular acetyl-CoA supply, robust mevalonate pathway flux, metabolic versatility, and GRAS status [13,14]. *Y. lipolytica* was successfully engineered to produce a UA titer of 254.3 mg/L [6], indicating that UA biosynthesis remains constrained by low cytochrome P450 efficiency, limited cofactor availability, and competition from endogenous sterol biosynthesis, thereby necessitating more integrated engineering strategies for improved production in *Y. lipolytica*.

Cytochrome P450 monooxygenases present inherent challenges for heterologous expression due to their reliance on efficient electron transfer from NADPH via CPR and adequate intracellular levels of FAD, FMN, heme, and iron [15,16]. In microbial hosts, cofactor availability is optimized for native metabolism rather than heterologous pathways, often limiting holoenzyme formation and reducing catalytic efficiency [17,18]. Plant-derived CYP716 enzymes additionally suffer from low expression, suboptimal membrane integration, restricted substrate access, and poor coupling [17,19,20]. As a result, P450 inefficiency constitutes a central bottleneck in microbial triterpenoid biosynthesis, requiring integrated strategies at both protein and cellular levels. Rational and semi-rational engineering of P450s, particularly structure-guided mutagenesis of substrate-binding residues and access tunnels, has effectively enhanced catalytic performance, substrate specificity, and turnover. Coupling these modifications with targeted cofactor reinforcement and systematic pathway optimization facilitates significant improvements in triterpenoid production [[21], [22], [23], [24], [25]].

In this study, we established a systematic engineering framework to enhance UA biosynthesis in *Y. lipolytica* by integrating rational enzyme engineering with cofactor and metabolic flux optimization. We first identified *CYP716A48* as the most efficient α-amyrin C-28 oxidase among several plant-derived candidates. Structure-guided reconstruction of its substrate-binding pocket, supported by molecular docking, enabled identification of key residues governing substrate access and catalytic efficiency. Rational mutagenesis yielded a superior Oe*CYP716A48*^D114Q/L211F^ variant with markedly enhanced UA production. To further alleviate P450 limitations, intracellular FAD, heme, and iron supply were systematically reinforced. Finally, multicopy integration of the optimized P450 module enabled efficient elimination of α-amyrin accumulation and maximized pathway flux, resulting in high-level UA production in fed-batch fermentation. Collectively, this work provides an informed strategy for overcoming cytochrome P450–associated limitations in triterpenoid biosynthesis and establishes *Y. lipolytica* as an efficient and scalable platform for sustainable UA production (Fig. 1).

## 2 Materials and methods

### 2.1 Strains and medium

The high α-amyrin–producing strain *Y. lipolytica* PO1f was used as the primary host in this study. Plasmid construction and amplification were performed in *Escherichia coli* JM109, which was cultured in Luria–Bertani (LB) medium containing 10 g/L tryptone, 5 g/L yeast extract, 10 g/L NaCl, and 100 μg/mL ampicillin at 37 °C for 16 h. Competent *E. coli* cells were prepared using a commercial kit (TaKaRa Bio Inc., Osaka, Japan). *Y. lipolytica* was grown in yeast peptone dextrose (YPD) medium composed of 10 g/L yeast extract, 20 g/L peptone, and 20 g/L glucose at 30 °C with shaking at 220 rpm. For yeast transformation, engineered cells were plated on yeast nitrogen base (YNB) medium containing 6.74 g/L YNB, 20 g/L glucose, and 5 g/L leucine to select for transformants.

### 2.2 Construction of plasmid and yeast transformation

Detailed information about strains and primers is listed in Tables S3,S4,S5. Amyrin synthasе (*CrMAS*) (GеnBank: JN991165) from *Catharanthus rosеus*, *MtCYP716A12* (GenBank: FN995112) from *Medicago truncatula*, *CaCYP716A83* (GenBank: KU878849) from *C. asiatica*, *CrCYP716AL1* (GenBank: JN565975) from *C. roseus*, *OeCYP716A48* (GenBank: AB706294.1) from *Olea europaea* and, *AtCPR1* (GenBank: BT008426.1) from *Arabidopsis thaliana* were synthеsizеd aftеr codon optimization by GENEWIZ Biotеch Co., Ltd. (Suzhou, China). The DNA sequences of the synthetic gene are listed in Table S7. Thе nativе gеnе was constructеd using thе *Y. lipolytica* gеnomе listes in Table S2. All plasmid construction was performed using Gibson assembly kit (Sangon Biotech Co., Ltd., Shanghai, China) [26] with the plasmid pYLXP’1 as a template. All assеmblеd plasmids wеrе confirmеd by Sangеr sеquеncing, and thе primеrs usеd wеrе synthеsizеd by Sangon Biotеch Co., Ltd. (Shanghai, China). Yeast transformation was performed using the Frozen-EZ Yeast Transformation II kit (Zymo Research, CA, USA), and cells were plated on YNB agar and incubated at 30 °C for 2–4 days. Transformants were confirmed by PCR using genomic DNA, followed by sequencing. For multicopy integration at the 26S ribosomal DNA (rDNA) loci, positive transformants were cultivated on uracil-dropout YNB plates, followed by curing of the URA3 selection marker as previously described [27]. Guide RNAs were designed using CHOPCHOP website. The integration sites used were non-essential in *Y.* *lipolytica* [28]. α-Amyrin and UA acid standards were purchased from Macklin (Shanghai, China).

### 2.3 Fеrmеntation of еnginееrеd yеast in shakе flasks and biorеactors

Single colonies of engineered *Y. lipolytica* strains were used to inoculate YPD medium and incubated for 24 h at 30 °C with shaking at 250 rpm. Then, 1 mL of each culture was transferred into 24 mL fresh YPD in 250 mL flasks and grown under the same conditions. Cultures were harvested after five days.

For enhanced amyrin production, the engineered strain was cultivated in a 5 L bioreactor using fed-batch fermentation. Seed cultures were prepared by inoculating single colonies into 10 mL YPD and incubating at 30 °C and 220 rpm for 24 h, followed by transfer into 200 mL YPD for an additional 24 h. The resulting culture was used to inoculate 2.2 L YPD in the bioreactor. Fermentation was conducted at 28 °C with pH maintained at 5.5 using ammonia solution, and dissolved oxygen controlled at 25% via an agitation cascade (300–900 rpm) and an aeration rate of 3.0 vvm. The initial glucose concentration was 40 g/L, and a concentrated glucose solution (500 g/L) was intermittently fed to maintain residual levels below 1 g/L. Upon depletion of the initial glucose, fed-batch feeding was initiated with a nutrient solution (100 g/L (NH_4_)_2_SO_4_, 35 g/L KH_2_PO_4_, 5 g/L MgSO_4_·7H_2_O) at 5–15 mL h^−1^.

### 2.4 Analytical methods

For α-amyrin analysis, 1 mL of *Y. lipolytica* culture broth was transferred into 2 mL grinding tubes (MP) and centrifuged at 12,000 rpm for 10 min. The supernatant was discarded, and the cell pellet was mixed with an equal volume of 0.5 mm glass beads and resuspended in 1 mL methanol. Cell disruption was performed using a FastPrep instrument for four cycles (6.0 m/s, 40 s per cycle). After lysis, samples were centrifuged again at 12,000 rpm for 10 min, and the supernatant was filtered through a 0.22 μm membrane prior to gas chromatography–mass spectrometry (GC–MS) analysis.

For UA extraction, ethanol was used instead of methanol following the same procedure. The resulting extracts were filtered through a 0.22 μm membrane and analyzed by high-performance liquid chromatography (HPLC).

α-Amyrin was analyzеd using a Shimadzu QP2010 GC-MS (Kyoto, Japan) еquippеd with an RTX-5 capillary column (30 m × 0.25 mm × 0.25 μm). Thе ovеn tеmpеraturе was rampеd at 20 °C/min to 280 °C, thеn at 40 °C/min to 300 °C and hеld for 10 min. Hеlium (1.0 mL/min) sеrvеd as thе carriеr gas, and 1 μL of samplе was injеctеd. Thе mass spеctromеtеr scannеd 35–500 *m/z* [29].

UA was dеtеctеd using HPLC on a C18 column (4.6 × 150 mm, 5 μm; Thеrmo Fishеr, Shanghai, China) with UV dеtеction at 210 nm and a column tеmpеraturе of 40 °C. Thе mobilе phasе consistеd of ultrapurе watеr, acеtonitrilе, and mеthanol in a ratio of 21:67:12 (v/v/v) [29].

### 2.5 Sеquеncе rеtriеval and multiplе sеquеncе alignmеnt

CYP716A cytochrome P450 amino acid sеquеncеs wеrе rеtriеvеd from thе GenBank databasе for rеprеsеntativе plant spеciеs (Tablе S1). Multiplе sеquеncе alignmеnt was pеrformеd using CLUSTAL Omеga with dеfault paramеtеrs. Sеquеncе consеrvation and alignmеnt visualization wеrе analyzеd using Jalviеw, whеrе consеrvation scorеs wеrе calculatеd basеd on amino-acid idеntity and physicochеmical similarity across alignеd sеquеncеs (Figs. S9 and S10).

### 2.6 Molecular docking

The three-dimensional structure of *CYP716A48* was retrieved from the AlphaFold Protein Structure Database (UniProt ID: A0A090AQE0), with an average pLDDT score of > 90.19. The pLDDT (predicted Local Distance Difference Test) score provides a per-residue confidence estimate ranging from 0 to 100, where higher values indicate greater reliability of the predicted atomic positions. The stereo-chemical quality of the obtained modeled structure of the query protein was verified using the MolProbity server (http://molprobity.biochem.duke.edu), which assesses the 3D structure and checks the quality of the predicted model. The three-dimensional structure of α-Amyrin (CID:73170) was downloaded from PubChem (https://pubchem.ncbi.nlm.nih.gov) in sdf format. Molecular docking of the ligand into the protein was performed using AutoDock Vina 1.2.0 [30], following standard docking protocols to predict ligand–protein interactions. The resulting protein–ligand complexes were assessed based on calculated binding energies, visualized and analyzed using the PyMOL 3.1 molecular graphics system, and residues within 5 Å of the ligand were considered for interaction analysis.

### 2.7 Computational tunnel engineering analysis

The heme cofactor, essential for *CYP716A48* catalytic activity as a cytochrome P450 monooxygenase, was incorporated using AlphaFill, which transfers experimentally resolved ligands from homologous crystal structures into predicted protein models through local structural alignment. A homologous cytochrome P450 crystal structure (PDB ID:2VE3) was used as the structural template, showing 46.15% sequence similarity with *CYP716A48*, and the transplanted heme group achieved a local RMSD of 3.08 Å, indicating reliable spatial positioning. Visual inspection using PyMOL confirmed correct orientation of the porphyrin ring within the conserved P450 heme-binding pocket and preservation of the canonical P450 motif, including the signature Cys ligand-binding region.

Tunnel analyses were conducted using CAVER 3.0 (PyMOL plugin) under constant parameters for wild-type and engineered *CYP716A48* variants: minimum probe radius 0.9 Å, shell depth 4 Å, shell radius 3 Å, clustering threshold 3.5 Å, maximum distance 3 Å, and desired radius 5 Å. From the dominant tunnel cluster for each variant, CAVER calculated five geometric descriptors: (1) bottleneck radius (Å), representing the narrowest constriction along the tunnel and the primary steric barrier for substrate entry to the heme catalytic center; (2) tunnel length (Å), representing the path distance from the heme iron center to the protein surface, where shorter distances indicate more efficient substrate diffusion pathways; (3) curvature (dimensionless), quantifying tunnel linearity, with lower values indicating straighter and more favorable transport pathways; (4) throughput (arbitrary units), representing overall transport efficiency as a composite function of tunnel geometry; and (5) cost (arbitrary units), representing the energetic and steric penalty associated with substrate passage through the tunnel. Tunnel surfaces and bottleneck constriction sites were visualized using PyMOL to qualitatively validate quantitative CAVER outputs and to identify key residues contributing to tunnel narrowing or bending regions targeted for rational mutagenesis. Because the heme iron center was fixed across all variants, while tunnel parameters directly reflected structural modifications, bottleneck radius, tunnel length, curvature, throughput, and cost metrics could be directly compared among wild-type and engineered *CYP716A48* variants to evaluate the structural and functional impact of designed mutations.

### 2.8 Statistical analysis

All experiments were conducted in three independent replicates. Results are expressed as mean values ± standard deviation (SD). Statistical analyses were performed using GraphPad Prism version 8 (GraphPad Software, San Diego, CA, USA). Group differences were assessed by one-way analysis of variance (ANOVA) followed by appropriate multiple comparison tests to determine statistical significance. A P-value <0.05 was considered statistically significant. Data visualization was carried out using OriginPro 2024 (OriginLab Corporation, Northampton, MA, USA). Reported numerical values were rounded to an appropriate number of significant figures consistent with the experimental variability and analytical precision.

## 3 Results

### 3.1 Screening of heterologous P450 candidates for ursolic acid production

**Fig. 2:**
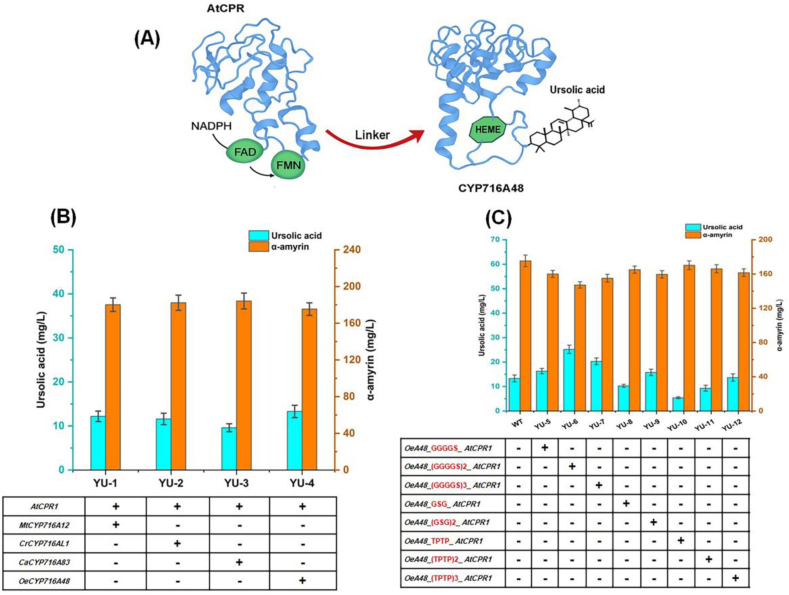
Engineering of *CYP716A48*–CPR fusion and evaluation of linker optimization for enhanced ursolic acid production. (A) Schematic representation of the self-sufficient fusion system constructed by linking cytochrome P450 reductase (*AtCPR*) to *CYP716A48* through a flexible peptide linker, enabling direct electron transfer from NADPH via FAD and FMN to the heme-containing *CYP716A48* for efficient oxidation of α-amyrin to ursolic acid. (B) Comparison of different plant-derived C-28 oxidases co-expressed with *AtCPR*. Ursolic acid (cyan) and α-amyrin (orange) titers were measured in strains expressing *MtCYP716A12*, *CrCYP716AL1*, *CaCYP716A83*, or *OeCYP716A48*. (C) Effects of fused linker designs on *CYP716A48*–*AtCPR* activity. Production levels of ursolic acid and α-amyrin are shown for the wild-type control (WT; no fusion enzyme, YU4) and engineered fusion variants (YU-5 to YU-12). All data represent the mean ± standard deviation (SD) of three independent biological replicates (P < 0.05).

To establish UA biosynthesis in *Y. lipolytica*, we employed the laboratory stock strain YU-0, which produced 197 ± 6 mg/L of α-amyrin under shake-flask conditions (Fig. S1, Table S5). Four plant cytochrome P450 monooxygenases, *MtCYP716A12*, *CrCYP716AL1*, *CaCYP716A83*, and *OeCYP716A48*, were individually integrated into the engineered parental strain YU-0 and co-expressed with *AtCPR1*, generating four recombinant strains designated YU-1 to YU-4, respectively. HPLC–UV and LC–Q-TOF/MS analyses confirmed successful UA biosynthesis in all engineered strains (Figs. S2, S3, S4, S6). Among them, strain YU-4 exhibited the highest performance, producing 13 ± 1 mg/L UA, while the α-amyrin titer decreased to 175 ± 7 mg/L (Fig. 2B, S8). *OeCYP716A48* demonstrated superior catalytic efficiency and produced the highest UA titer among the evaluated P450 variants, and was therefore selected for subsequent optimization studies.

To enhance the catalytic efficiency of *OeCYP716A48* in *Y. lipolytica*, we engineered a series of fusion enzymes by linking *OeCYP716A48* to its redox partner *AtCPR1* using peptide linkers with varying lengths and flexibilities (Fig. 2A). The resulting variants were expressed in strain YU-0 and evaluated for UA production. UA production varied substantially among the fusion constructs (Fig. 2C–S8). Among the tested linkers, the flexible (GGGGS)_2_ linker exhibited the best performance, resulting in the highest UA titer of 25 ± 2 mg/L, while the α-amyrin titer decreased to 147 ± 4 mg/L in the YU-6 strain, corresponding to an approximately 92% increase relative to the YU-4 strain. Nevertheless, UA titers remained relatively low, suggesting that additional metabolic and protein-engineering strategies will be necessary to further improve enzyme performance.

### 3.2 Engineering *OeCYP716A48* for enhanced ursolic acid biosynthesis

**Fig. 3:**
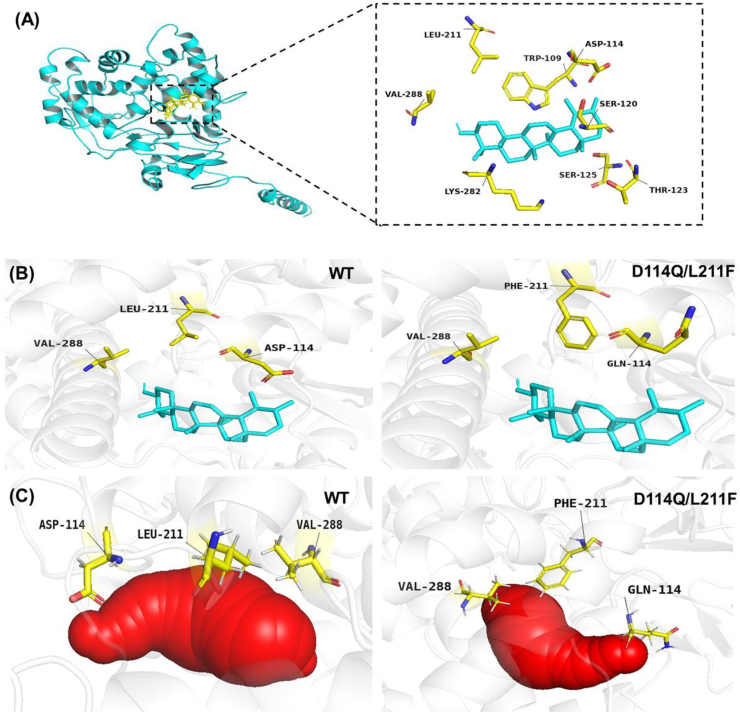
Structural analysis of the *OeCYP716A48* active site and effects of D114Q/L211F mutations on substrate binding. (A) Overall three-dimensional structure of *OeCYP716A48* showing the substrate-binding pocket. The protein backbone is displayed as a cyan cartoon, while the bound ligand is shown in stick representation (cyan). Key residues surrounding the active site are highlighted in yellow, and an enlarged view illustrates their spatial arrangement relative to the ligand. (B) Close-up views of the active site in the wild-type (WT) and mutant (D114Q/L211F) enzymes. (C) The substrate access tunnel is visualized, highlighting residues lining the tunnel near the catalytic site. In the WT, bulky side chains partially restrict the tunnel, while the D114Q/L211F mutations expand and smooth the tunnel architecture, facilitating improved substrate access and accommodation.

A multiple sequence alignment of CYP716A homologs from diverse plant species was performed to identify residues with potential functional relevance (Table S1). The analysis revealed strong conservation within core catalytic regions such as the heme-binding cysteine loop, whereas substrate recognition sites (SRSs) and surface-exposed regions exhibited higher variability (Fig. S10–S11). These variable positions were considered potential targets for protein engineering. Among the analyzed homologs, *OeCYP716A48* showed the highest sequence similarity to *CrCYP716AL1* (83.92%), indicating close evolutionary relatedness (Fig. S9). To define substrate interactions, α-amyrin was docked into the *OeCYP716A48* active site. The substrate occupied a predominantly hydrophobic pocket, with residues A107, W108, W109, V113, D114, F117, S120, T123, S124, S125, E128, L211, D281, K282, G285, L286, V288, G289, and R424 located within 5 Å (Fig. 3A). Integration of docking results with conservation analysis revealed that Several residues, including W108, W109, F117, E128, G285, G289, and R424, were fully conserved, indicating essential roles in catalysis or structural integrity, where mutations are likely to disrupt enzyme function. Residues exhibiting high conservation, A107, D281, K282, L286, V288, S124, and S125, contribute to substrate recognition and structural stability and may tolerate subtle modifications. In contrast, residues with moderate conservation, including D114, S120, T123, and L211, display partial variability, suggesting involvement in substrate binding while maintaining sufficient flexibility for functional testing.

**Fig. 4:**
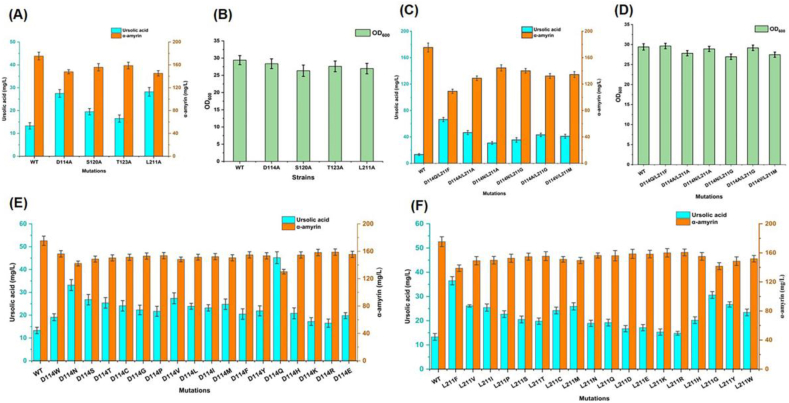
Structure-guided mutagenesis of *CYP716A48* improves ursolic acid production. (A) Ursolic acid and α-amyrin production in single-site mutants (D114A, S120A, T123A, and L211A) compared with the wild-type strain (YU4). (B) Cell growth (OD_600_) of the strains shown in panel (A). (C) Ursolic acid and α-amyrin production in combinatorial mutants generated based on the best single-site variants. (D) Cell growth (OD_600_) of the strains shown in panel (C), confirming. (E) Saturation mutagenesis at residue D114 showing the effect of amino-acid substitutions on ursolic acid production. (F) Saturation mutagenesis at residue L211. Ursolic acid (cyan) and α-amyrin (orange) titers. All data represent the mean ± standard deviation (SD) of three independent biological replicates (P < 0.05).

To assess the functional roles of selected residues, alanine-scanning mutagenesis was performed, and the resulting variants were co-expressed with *AtCPR1* in the YU-0 strain. Among the tested mutants, D114A and L211A showed the most pronounced effects on UA production (Fig. 4A and B). The D114A variant produced 27 ± 2 mg/L UA, representing a 2-fold increase compared to the wild type, with a reduction in α-amyrin to 148 ± 4 mg/L. Similarly, L211A produced 28 ± 2 mg/L UA (2.2-fold increase), with α-amyrin reduced to 145 ± 5 mg/L. Based on these results, residues D114 and L211 were subjected to site-saturation mutagenesis. Among the resulting variants (Fig. 4E, F, S7), D114Q exhibited the highest improvement, producing 45 ± 3 mg/L UA (3.5-fold increase), with α-amyrin reduced to 130 ± 3 mg/L. The L211F variant produced 37 ± 2 mg/L UA (2.8-fold increase), with α-amyrin at 139 ± 5 mg/L. To further enhance catalytic performance, beneficial mutations were combined (Fig. 4C and D). The double mutant D114Q/L211F produced 66 ± 3 mg/L UA, corresponding to a 5-fold increase compared to the wild type, with α-amyrin reduced to 109 ± 3 mg/L.

The substrate access tunnel of *OeCYP716A48* was analyzed using Caver to evaluate the effects of the D114Q/L211F double mutation on substrate accessibility. Compared with the wild-type enzyme, the D114Q/L211F variant showed an increased tunnel throughput from 0.71 to 0.76, indicating enhanced substrate passage. Meanwhile, the tunnel cost decreased from 0.33 to 0.27, suggesting a more energetically favorable pathway for substrate diffusion. The bottleneck radius expanded from 1.12 Å to 1.52 Å, reflecting a wider constriction that may better accommodate bulky substrates. In addition, the tunnel length decreased from 18.37 Å to 16.87 Å, while the curvature slightly increased from 1.14 to 1.25, indicating a shorter yet slightly more curved pathway toward the active site that could facilitate substrate access (Fig. 3B and C). Together, these structural changes suggest that the D114Q/L211F mutations improve substrate access by enlarging the tunnel entrance, reducing steric and energetic barriers, and facilitating substrate alignment, which likely contributes to the observed enhancement in enzymatic activity. The strain YU-13, harboring *OeCYP716A48* ^D114Q/L211F^, was selected for further studies.

### 3.3 Engineering the FAD biosynthesis pathway to enhance *OeCYP716A48* activity

**Fig. 5:**
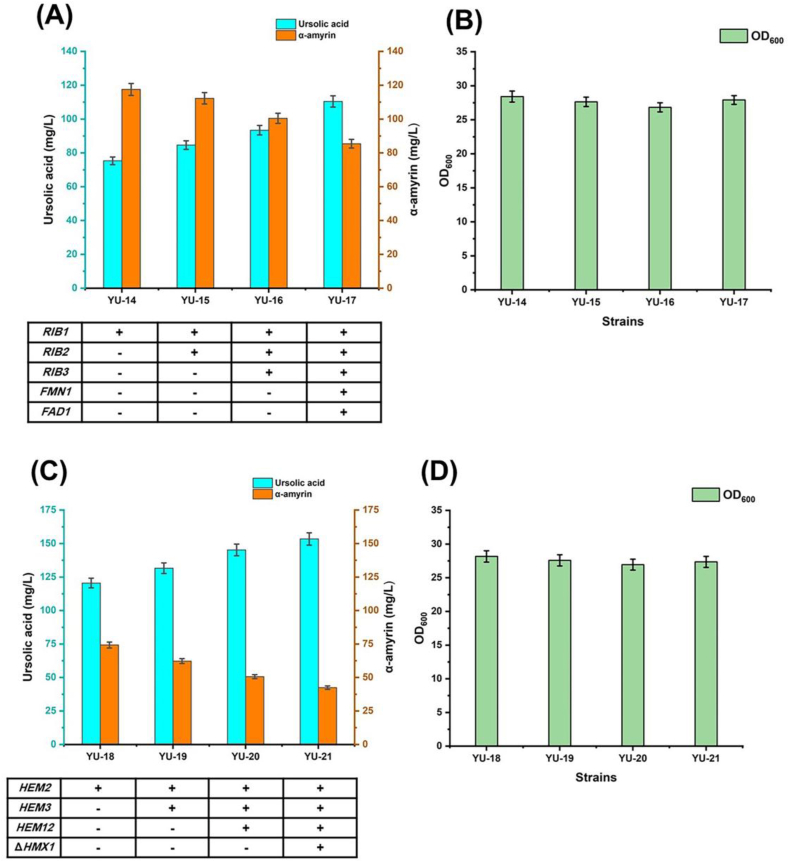
Systematic engineering of riboflavin and heme biosynthesis enhances ursolic acid production. (A) Effect of stepwise overexpression of riboflavin biosynthetic genes (*RIB1*, *RIB2*, *RIB3*, *FMN1* and *FAD1*) on ursolic acid and α-amyrin production. (B) Cell growth (OD_600_) of strains YU-14 to YU-17. (C) Effect of reinforcing heme biosynthesis via overexpression of *HEM2*, *HEM3*, *HEM12*, and deletion of *HMX1* on ursolic acid and α-amyrin production. (D) Cell growth (OD_600_) of strains YU-18 to YU-21. Ursolic acid (cyan) and α-amyrin (orange) titers. All data represent the mean ± standard deviation (SD) of three independent biological replicates (P < 0.05).

To improve the catalytic efficiency of *OeCYP716A48* and enhance the conversion of α-amyrin to UA in *Y. lipolytica*, we engineered the endogenous riboflavin–FAD biosynthesis pathway to increase intracellular FAD(H)_2_ availability. Specifically, key genes involved in flavin biosynthesis and conversion, including *RIB1*, *RIB2*, *RIB3*, and *FMN1*, were systematically overexpressed to strengthen cofactor supply and support *OeCYP716A48*-dependent oxidation reactions (Fig. 1, Fig. 5). Overexpression of *RIB1*, encoding the first committed enzyme in riboflavin biosynthesis, enhanced precursor flux toward flavin cofactors and increased UA production to 75 ± 2 mg/L, confirming cofactor limitation in YU-13 strain. Subsequent co-expression of *RIB2* and *RIB3*, which catalyze downstream steps in riboflavin biosynthesis, further improved UA titer to 93 ± 3 mg/L, accompanied by a concomitant reduction in α-amyrin accumulation, indicating more efficient substrate conversion. Finally, overexpression of *FMN1* and *FAD1*, responsible for converting riboflavin to FMN and supporting FAD biosynthesis, further strengthened cytosolic FAD(H)_2_ supply and increased UA production to 110 ± 4 mg/L. Collectively, these results demonstrate that systematic reinforcement of the native riboflavin–FAD biosynthesis pathway significantly improved P450 catalytic performance and UA biosynthesis in the engineered strain.

### 3.4 Optimization of heme supply and iron uptake system to improve *OeCYP716A48* activity

The catalytic efficiency of cytochrome P450 enzymes is critically dependent on intracellular heme availability, as heme serves as the essential cofactor at the enzyme's active site, enabling oxygen activation and electron transfer during catalysis. Insufficient heme limits holoenzyme formation, thereby reducing overall P450 activity [31,32]. To enhance *OeCYP716A48*-mediated oxidation and boost UA production, we systematically engineered the endogenous heme biosynthetic pathway in *Y. lipolytica* (Fig. 1, Fig. 5). Overexpression of *HEM2* increased UA production to 120 ± 4 mg/L, indicating that strengthening early heme biosynthesis enhances precursor supply. Further Overexpression of *HEM3* increased UA titer to 132 ± 4 mg/L, suggesting increased flux through the tetrapyrrole pathway, with the greater improvement compared to *HEM2* indicating stronger flux control at this step under the tested conditions. Reinforcing downstream steps via *HEM12* overexpression further increased UA production to 145 ± 4 mg/L, demonstrating the importance of optimizing late-stage heme biosynthesis. Finally, deletion of *HMX1* increased UA titer to 153 ± 5 mg/L, likely by reducing heme degradation and enhancing intracellular heme retention, thereby improving *OeCYP716A48* catalytic efficiency.

Since heme biosynthesis depends on intracellular iron availability, we engineered the high-affinity iron uptake system in *Y. lipolytica* to enhance *OeCYP716A48* activity and UA production (Fig. 1, Fig. 5). Overexpression of *FTR1* increased UA production to 166 ± 5 mg/L, indicating that iron transport capacity limited P450-dependent biosynthesis. Further upregulation of *FET3*, which forms a functional complex with *FTR1*, increased UA titer to 178 ± 5 mg/L, suggesting improved efficiency of iron translocation and sustained heme supply. Finally, overexpression of *FRE1*, the plasma membrane ferric reductase, further increased UA production to 187 ± 6 mg/L, demonstrating the importance of extracellular ferric reduction in iron assimilation and subsequent heme biosynthesis.

### 3.5 Fed-batch fermentation in 5-L bioreactor

**Fig. 6:**
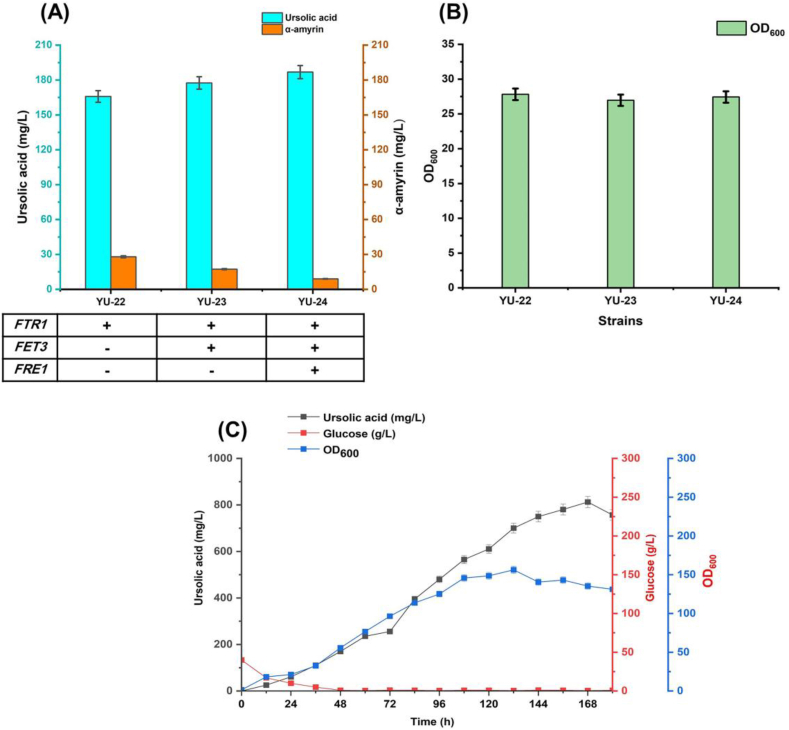
Enhancement of ursolic acid production by engineering the high-affinity iron uptake system and 5L fermentation. (A) Effect of stepwise overexpression of iron uptake genes (*FTR1*, *FET3*, and *FRE1*) on ursolic acid and α-amyrin production. All data represent the mean ± standard deviation (SD) of three independent biological replicates (P < 0.05). (B) Cell growth (OD_600_) of strains YU-22 to YU-24 (C) Time-course fermentation profile of the best-performing strain (YU-25). Ursolic acid production, glucose consumption, and cell growth (OD_600_) were monitored during cultivation. All data represent the mean ± standard deviation (SD) of three independent biological replicates (P < 0.05).

To further enhance UA production by redirecting metabolic flux, *CrMAS*, *OeCYP716A48*^D114Q/L211F^, and *AtCPR1* were integrated in multiple copies at the rDNA locus in strain YU-24. The resulting transformants were screened for UA production, among which strain YU-25 exhibited the highest titer (205 ± 7 mg/L), with α-amyrin no longer detectable. These results suggest efficient flux redirection and stable multi-copy expression (Fig. S5, Table S6) [[33], [34], [35]]. Fed-batch fermentation was subsequently performed in a 5-L bioreactor to evaluate the production capacity of the YU-25 strain (Fig. 6C). UA reached its maximum titer at 168 h, yielding 813 ± 24 mg/L, representing the highest ursolic acid production reported to date in *Y. lipolytica*. Based on the total glucose consumption and the estimated final working volume, the overall yield was approximately 3.1 mg/g glucose. The average volumetric productivity reached 4.8 mg/L/h (116.2 mg/L/day) at 168 h, while the substrate consumption rate during fermentation was approximately 1.5 g/L/h. These results validate the effectiveness of our enzyme and pathway engineering strategies in overcoming key metabolic bottlenecks and enabling high-level triterpenoid production.

## 4 Discussion

In this study, an efficient α-amyrin–producing *Y. lipolytica* chassis was used as the starting platform to investigate downstream limitations in UA biosynthesis. Because the parental strain already accumulated a large α-amyrin pool, the terminal cytochrome P450–dependent oxidation step became the dominant bottleneck restricting UA formation. Screening of four CYP716A monooxygenases identified *OeCYP716A48* as the most efficient enzyme, yet UA production remained low while α-amyrin accumulated, indicating insufficient catalytic turnover. Similar limitations have been widely reported in microbial triterpenoid biosynthesis, where plant P450 enzymes often display low activity due to inefficient electron transfer, poor coupling with CPR, and limited cofactor availability [6,10,12,18]. Fusion of *OeCYP716A48* with *AtCPR1* confirmed that spatial organization between the monooxygenase and its reeducates strongly influences catalytic performance, and the flexible (GGGGS)_2_ linker significantly increased UA production, consistent with previous reports showing that P450–CPR fusion enhances electron transfer efficiency and improves oxidation reactions in engineered hosts [15,17,19,36]. Nevertheless, the moderate improvement indicated that intrinsic structural constraints within the enzyme still limited substrate conversion, necessitating further protein engineering.

Structure-guided mutagenesis of *OeCYP716A48* demonstrated that active-site architecture and substrate-access geometry play critical roles in determining catalytic efficiency. Sequence conservation analysis combined with molecular docking allowed identification of moderately conserved residues suitable for engineering, and alanine scanning revealed D114 and L211 as key functional positions. Site-saturation mutagenesis generated the D114Q and L211F variants, and their combination further increased UA production more than 5-fold compared with the wild-type enzyme. Tunnel analysis showed that the double mutant exhibited an enlarged bottleneck radius, reduced energetic cost, and increased predicted substrate throughput, suggesting that improved diffusion of α-amyrin toward the heme center contributed to enhanced catalytic turnover. Similar strategies have been successfully used to improve cytochrome P450 activity through remodeling of substrate-binding residues and access tunnels, which can increase catalytic efficiency without disrupting protein folding [21,22,24,25]. These results confirm that structural constraints rather than expression level alone often determine the performance of plant P450 enzymes in microbial cell factories.

Because P450 catalysis depends on efficient electron transfer through flavin cofactors, cofactor availability became limiting after improving enzyme structure. Reinforcement of the riboflavin–FAD biosynthesis pathway by overexpressing *RIB1*, *RIB2*, *RIB3*, *FMN1*, and *FAD1* progressively increased UA production, demonstrating that intracellular flavin supply restricts CPR-mediated electron transfer under high-flux conditions. Previous studies have shown that insufficient FAD and FMN levels reduce coupling efficiency between CPR and P450, leading to poor catalytic performance in heterologous systems [15,17,18]. The stepwise improvement observed here indicates that cofactor limitation emerges as a secondary bottleneck once the primary catalytic constraint is relieved, emphasizing the importance of coordinated optimization of enzyme engineering and cellular metabolism. In addition to flavin cofactors, cytochrome P450 monooxygenases require heme as an essential prosthetic group for oxygen activation and electron transfer. Engineering the tetrapyrrole pathway has been widely employed to enhance hemoprotein activity in yeast, as efficient holoenzyme formation requires sufficient intracellular heme [37,38]. Previous studies have shown that overexpression of heme genes, combined with deletion of the heme oxygenase genes, significantly increases intracellular heme levels, with reported enhancements ranging from 4-fold to over 40-fold depending on the optimization strategy [32,38]. overexpression of *HEM2*, *HEM3*, and *HEM12* with *HMX1* knockout increased Asiatic acid production in *S. cerevisiae* by 3.19-fold [39], while overexpression of the same genes combined with *ROX1* and *HMX1* knockout enhanced chelerythrine production in *S. cerevisiae* by 68.6% [40]. In our study, UA production increased 1.4-fold, suggesting that reduced heme degradation and enhanced intracellular heme retention, thereby improving *OeCYP716A48* catalytic efficiency. Heme biosynthesis in P450-dependent pathways requires iron incorporation, and limited iron availability can become a bottleneck in highly engineered yeast strains. Previous studies have shown that overexpression of high-affinity iron uptake components, such as *FIT2*, *FET3*, and *FTR1*, increased the production of 11, 20-dihydroxyferruginol in *S. cerevisiae* by 9.6% [17,41,42]. In our study, overexpression of *FRE1*, *FET3*, and *FTR1* further increased UA production by 22%, suggesting that coordinated optimization of iron assimilation, heme biosynthesis, and cofactor supply can substantially enhance *OeCYP716A48* catalytic efficiency.

After resolving catalytic and cofactor limitations, pathway amplification was performed to increase metabolic flux toward UA. Multicopy integration of *CrMAS*, *OeCYP716A48*^D114Q/L211F^, and *AtCPR1* at rDNA loci significantly increased UA production and eliminated detectable α-amyrin accumulation, indicating efficient precursor conversion. Fed-batch fermentation in a 5-L bioreactor validated this strategy, yielding 813 ± 24 mg/L UA, the highest reported in *Y. lipolytica*. While higher UA titers have been reported in *S. cerevisiae*, *Y. lipolytica* offers advantages such as strong acetyl-CoA supply, high lipid metabolism capacity, and robustness toward hydrophobic metabolites, making it a promising chassis for triterpenoid production [6,43]. The large improvement obtained in this work demonstrates that the main limitations in UA biosynthesis arise from P450 inefficiency, insufficient cofactor supply, and restricted heme formation rather than precursor availability. The sequential resolution of these bottlenecks highlights that achieving industrially relevant titers of P450-dependent metabolites requires coordinated optimization at multiple levels, including enzyme structure, electron transfer, cofactor metabolism, prosthetic-group biosynthesis, and metal homeostasis. This work demonstrates that main limitations in UA biosynthesis arise from P450 inefficiency, insufficient cofactor supply, and restricted heme formation rather than precursor availability. Structure-guided remodeling of *OeCYP716A48* enhanced substrate accessibility and catalytic efficiency, while reinforcement of flavin biosynthesis, heme production, and iron uptake ensured sufficient cofactor supply. Multicopy pathway integration balanced metabolic flux, enabling efficient conversion of α-amyrin to UA. These strategies are broadly applicable to other oxidosqualene-derived natural products, providing a generalizable approach to overcome intrinsic limitations of plant P450 enzymes in microbial hosts and advance scalable triterpenoid production.

## 5 Conclusion

This study establishes *Y. lipolytica* as an efficient platform for microbial UA production through integrated enzyme engineering, pathway balancing, and cofactor optimization. By systematically addressing the primary bottleneck in cytochrome P450–dependent oxidation, *OeCYP716A48* was identified as the most effective C-28 oxidase. Structure-guided engineering and CPR fusion optimization significantly enhanced catalytic efficiency and substrate conversion. Reinforcement of the riboflavin–FAD pathway improved electron transfer capacity, while engineering of the heme biosynthesis pathway and high-affinity iron uptake system demonstrated that tetrapyrrole flux and iron availability are critical for functional P450 activity. Multicopy integration of the optimized module enabled efficient coupling between α-amyrin supply and downstream oxidation, eliminating precursor accumulation and maintaining stable metabolic flux toward UA. These combined strategies yielded 813 ± 24 mg/L UA in fed-batch fermentation, the highest titer reported in *Y. lipolytica*, confirming that efficient triterpenoid biosynthesis requires coordinated optimization of enzyme activity, cofactor supply, and heme formation. This work provides a generalizable framework for improving cytochrome P450–dependent pathways and can be extended to other oxidosqualene-derived triterpenoids, supporting scalable and sustainable microbial production of high-value natural products.

## CRediT authorship contribution statement

**Hany Elsharawy:** Writing – review & editing, Writing – original draft, Visualization, Validation, Conceptualization. **Qian He:** Investigation. **Weizhu Zeng:** Investigation. **Jingwen Zhou:** Writing – review & editing, Validation, Supervision, Funding acquisition.

## Declaration of competing interest

The authors declare that they have no known competing financial interests or personal relationships that could have appeared to influence the work reported in this paper.
