# Supplementary material for: Systematic enzyme and cofactor engineering for efficient ursolic acid biosynthesis in Yarrowia lipolytica
Source: Synth Syst Biotechnol. 2026 Jul 17;16:88–98. doi: 10.1016/j.synbio.2026.05.012 (PMC13400399; doi:10.1016/j.synbio.2026.05.012)
Supplement: Multimedia component 1 [file mmc1.docx]

**Supplementary material**


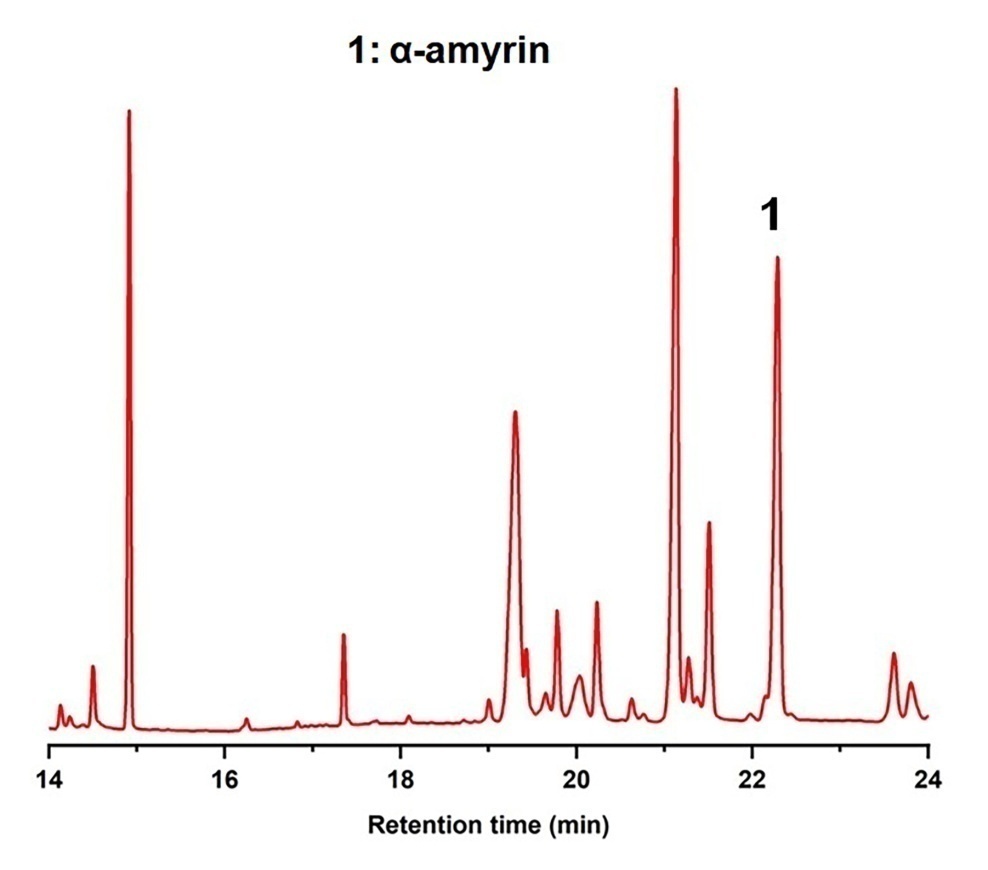


**Figure S1. GC–MS chromatogram of the extraction broth obtained from the** **YU-0 strain culture**, which was engineered in our laboratory and used as the starting strain for this study. Peak 1 indicates α-amyrin based on matching retention time with the standard.


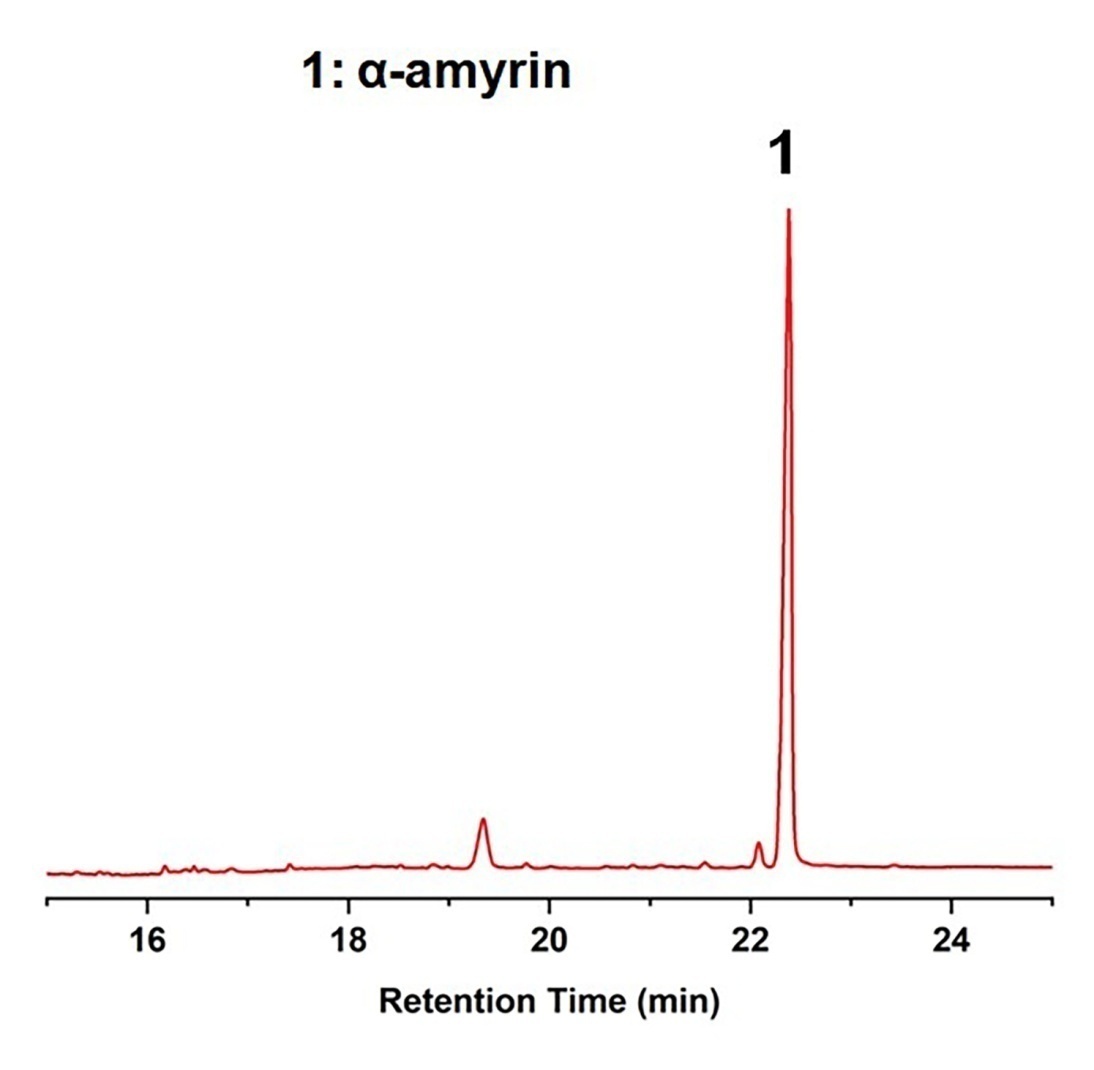


**Figure S2. GC–MS chromatogram of the α-amyrin standard.**


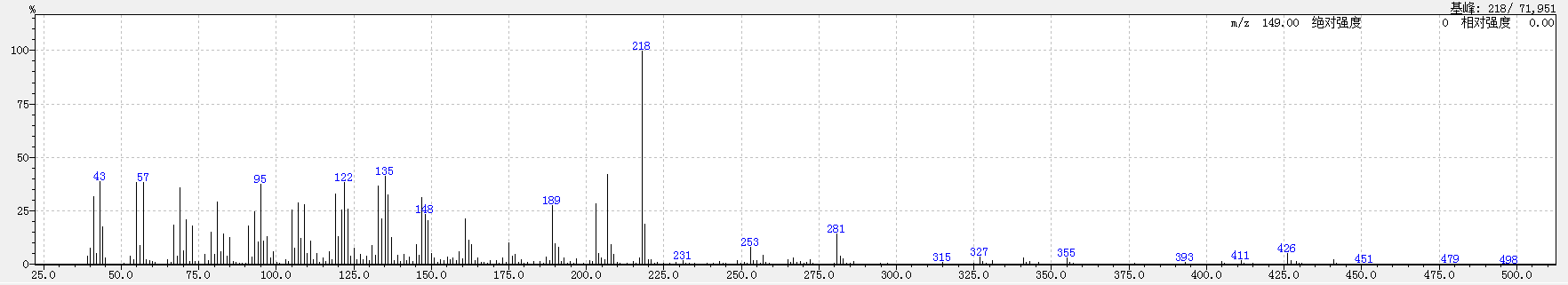


**Figure S3. Mass spectrum of α-amyrin in GC-MS.**


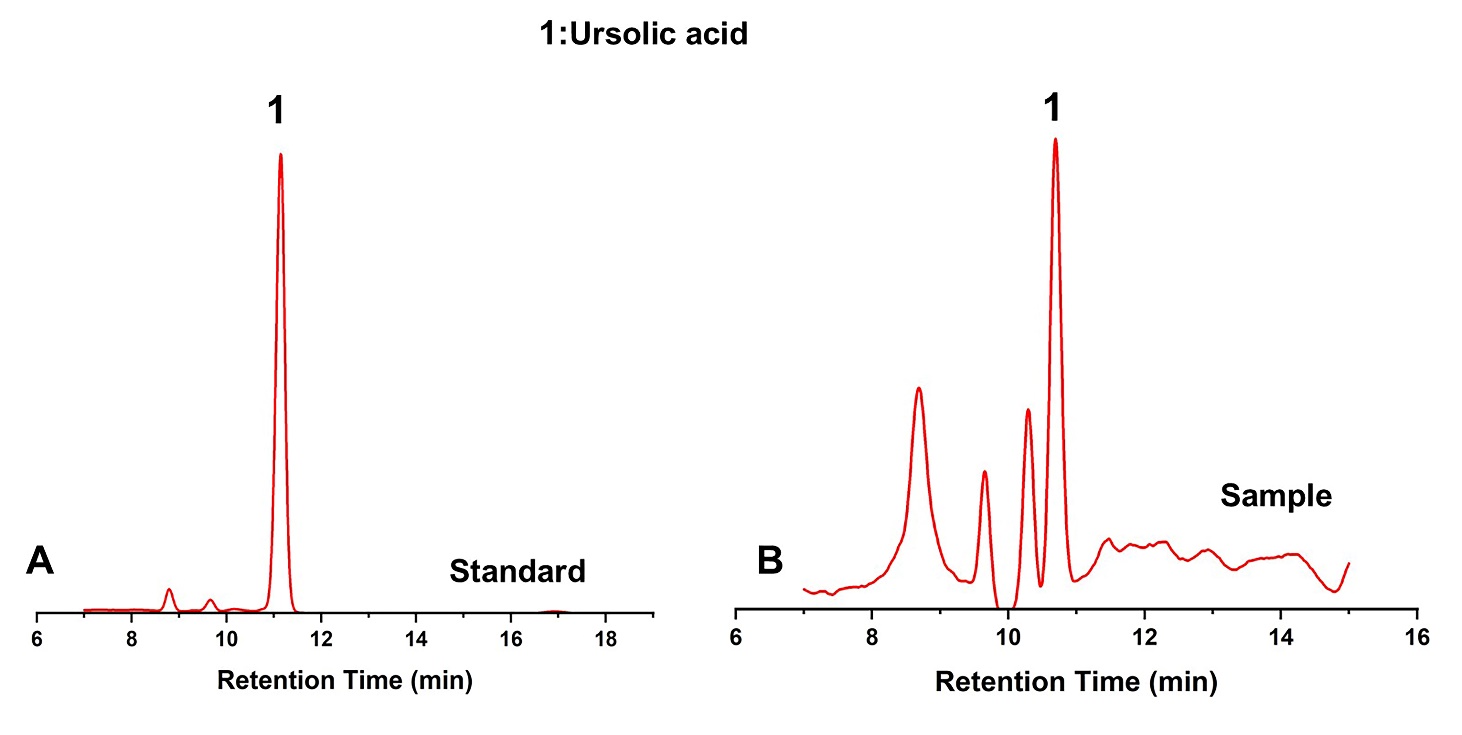


**Figure S4. HPLC analysis of ursolic acid.**

**(A) Chromatogram of the ursolic acid standard. (B) Chromatogram o the sample extract. Peak 1 indicates ursolic acid based on matching retention time with the standard.**

**
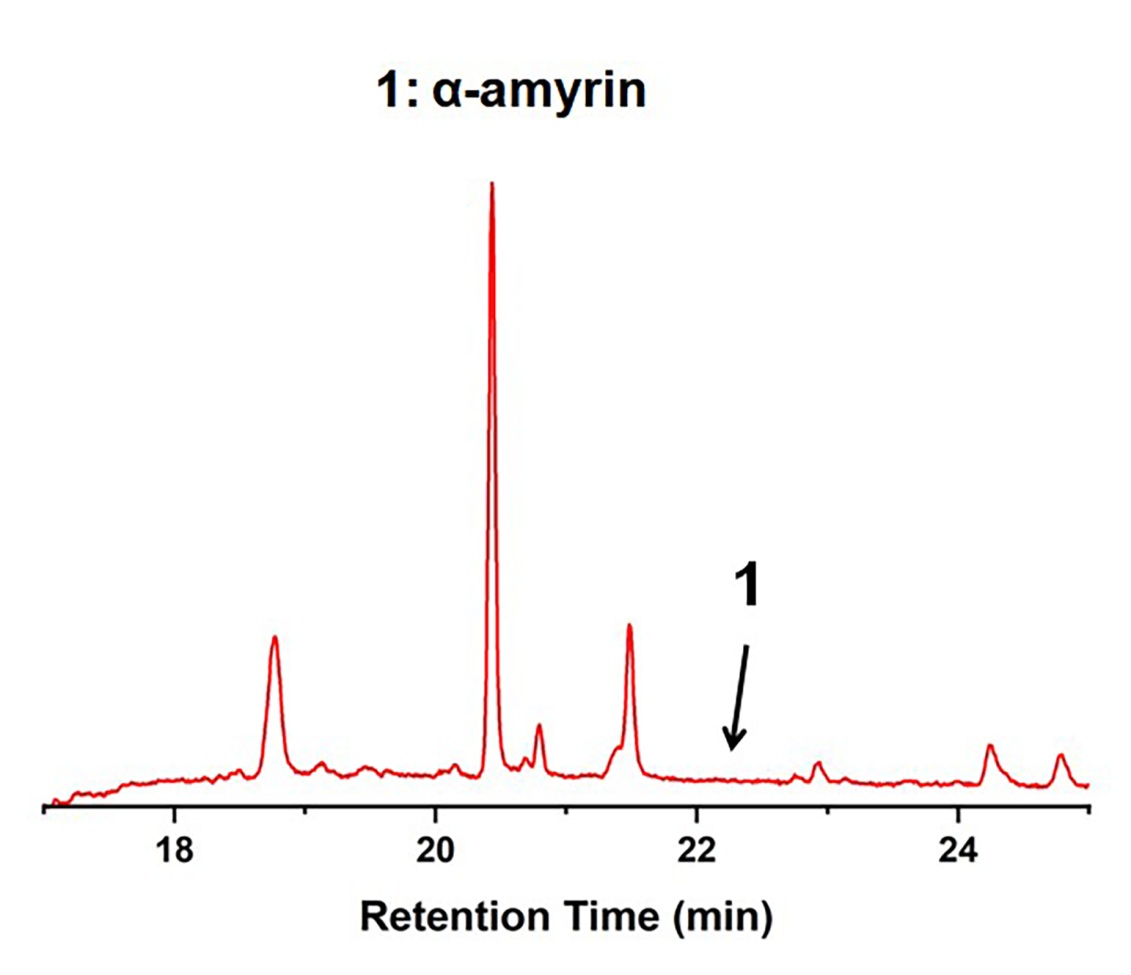
**

**Figure S5. GC–MS chromatogram of the extracted broth from the YU-25 strain culture. No detectable α-amyrin intermediate was observed, indicating efficient metabolic flux channeling toward the ursolic acid biosynthetic pathway.**


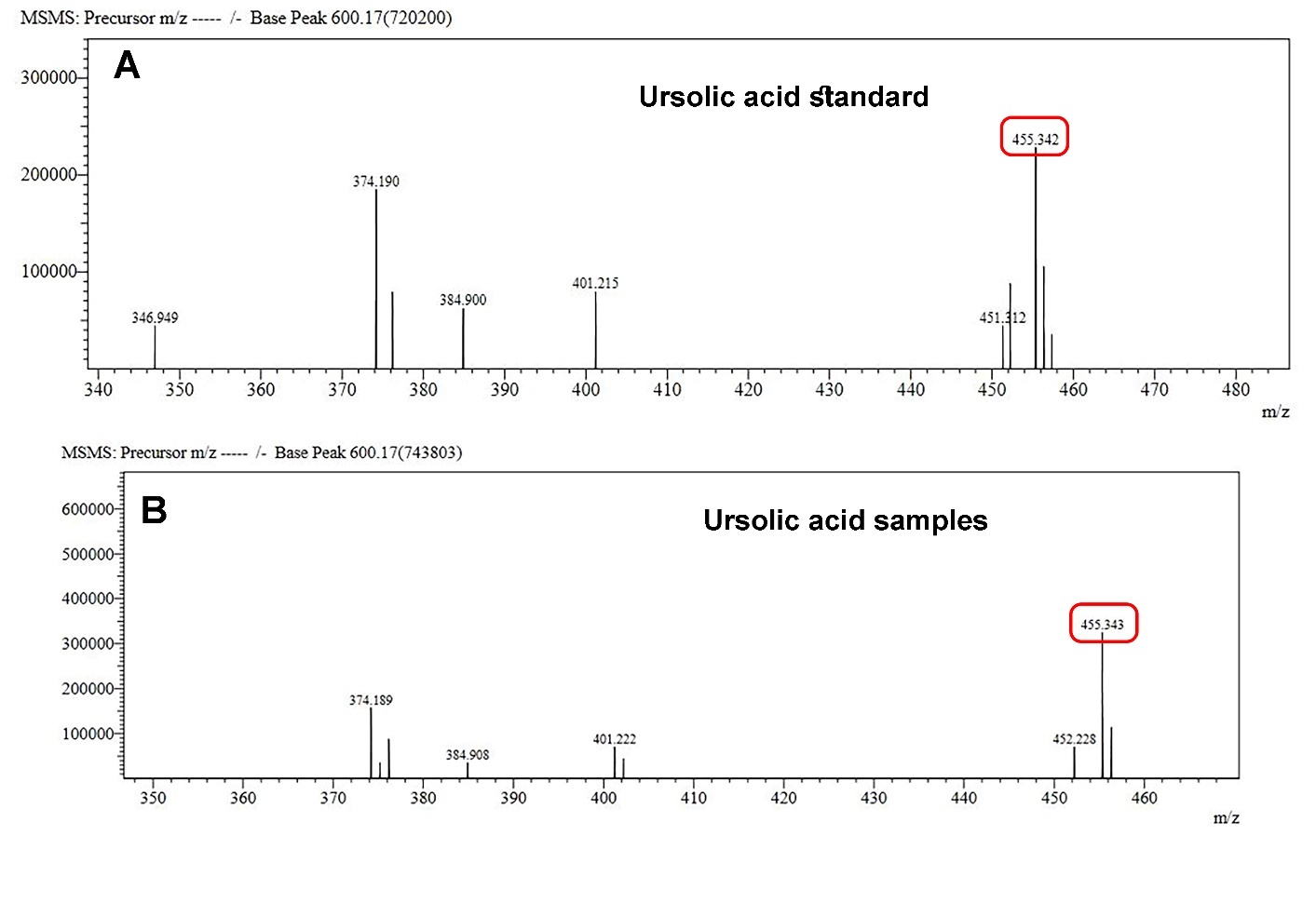


**Figure S6. LC-Q-TOF/MS spectra of ursolic acid standard (A) and samples (B).**


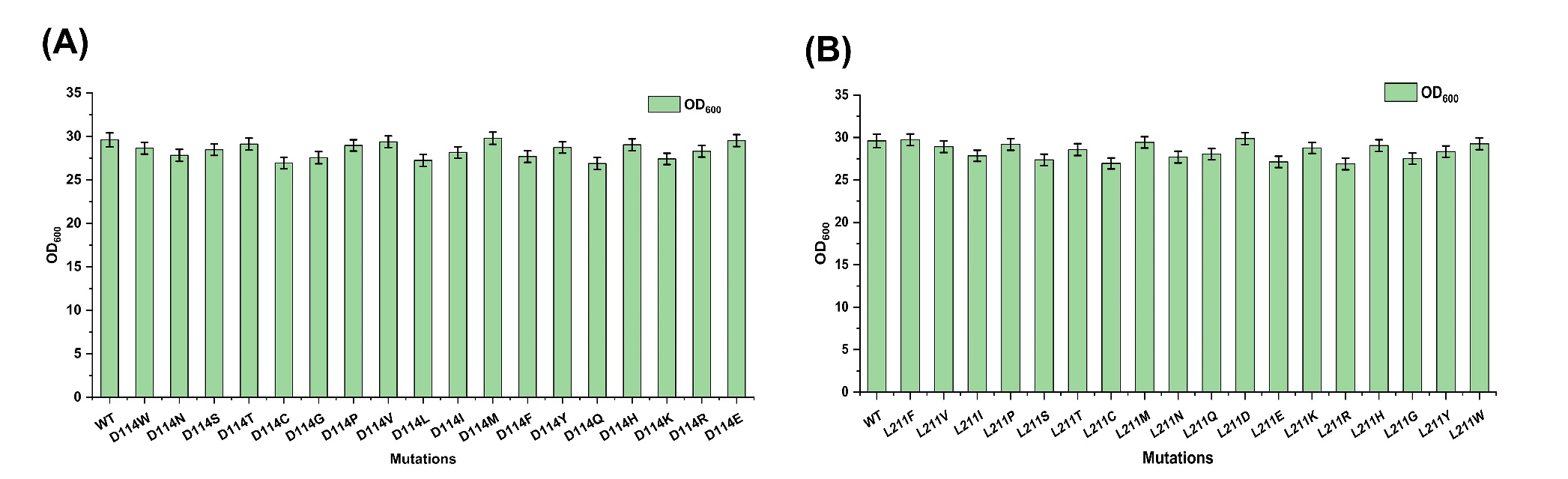


**Figure S7. Effect of saturation mutagenesis on cell growth.**

(A) Saturation mutagenesis at the D114 site. density (OD₆₀₀; green bars).(B) Saturation mutagenesis at the L211 site. Bars represent the mean of three independent experiments, and error bars indicate the standard deviation


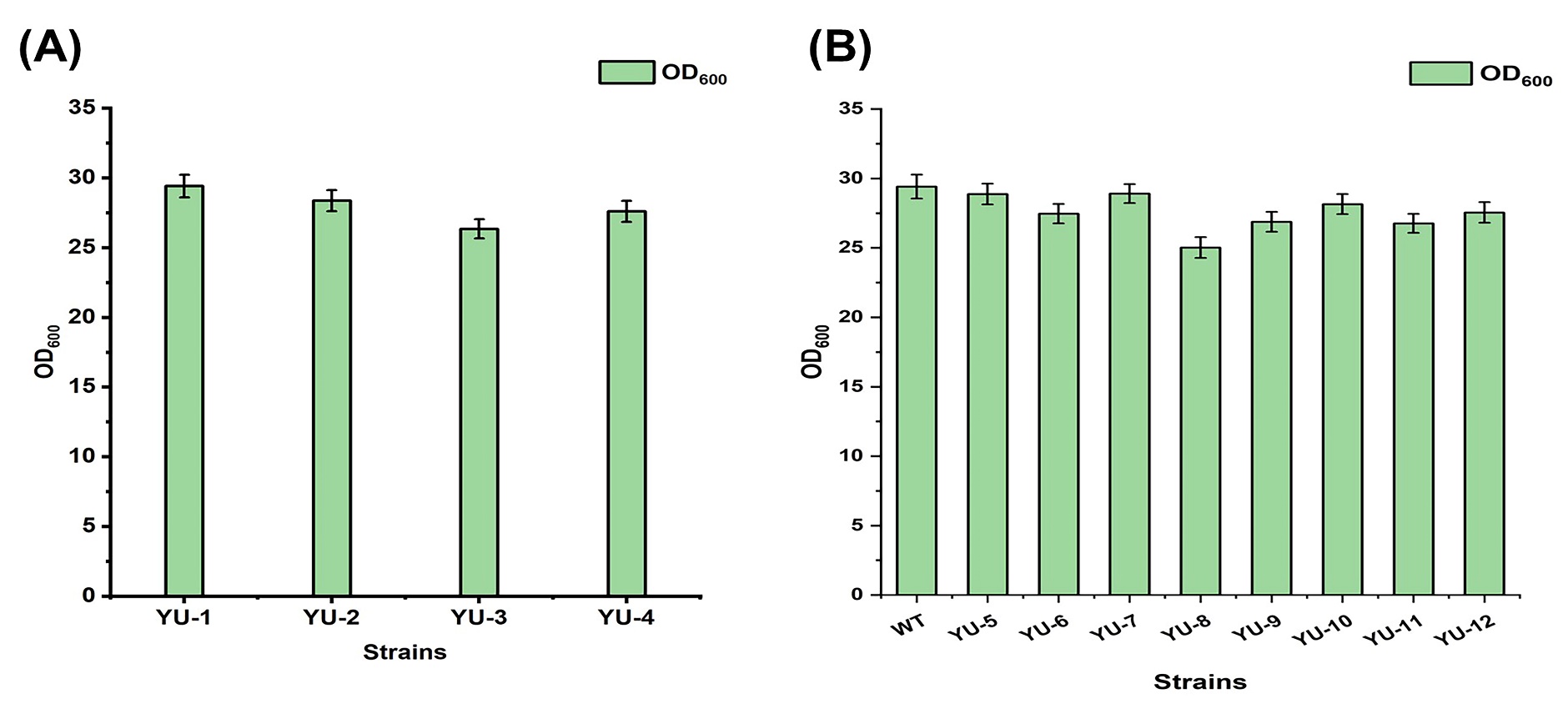


**Figure S8. Growth evaluation of engineered *Yarrowia lipolytica* strains.**(A) Cell growth of strains YU-1 to YU-4 measured as optical density at 600 nm (OD₆₀₀).
(B) Comparison of cell growth between the wild-type (WT) strain and engineered strains YU-5 to YU-12. Bars represent the mean of three independent experiments, and error bars indicate the standard deviation


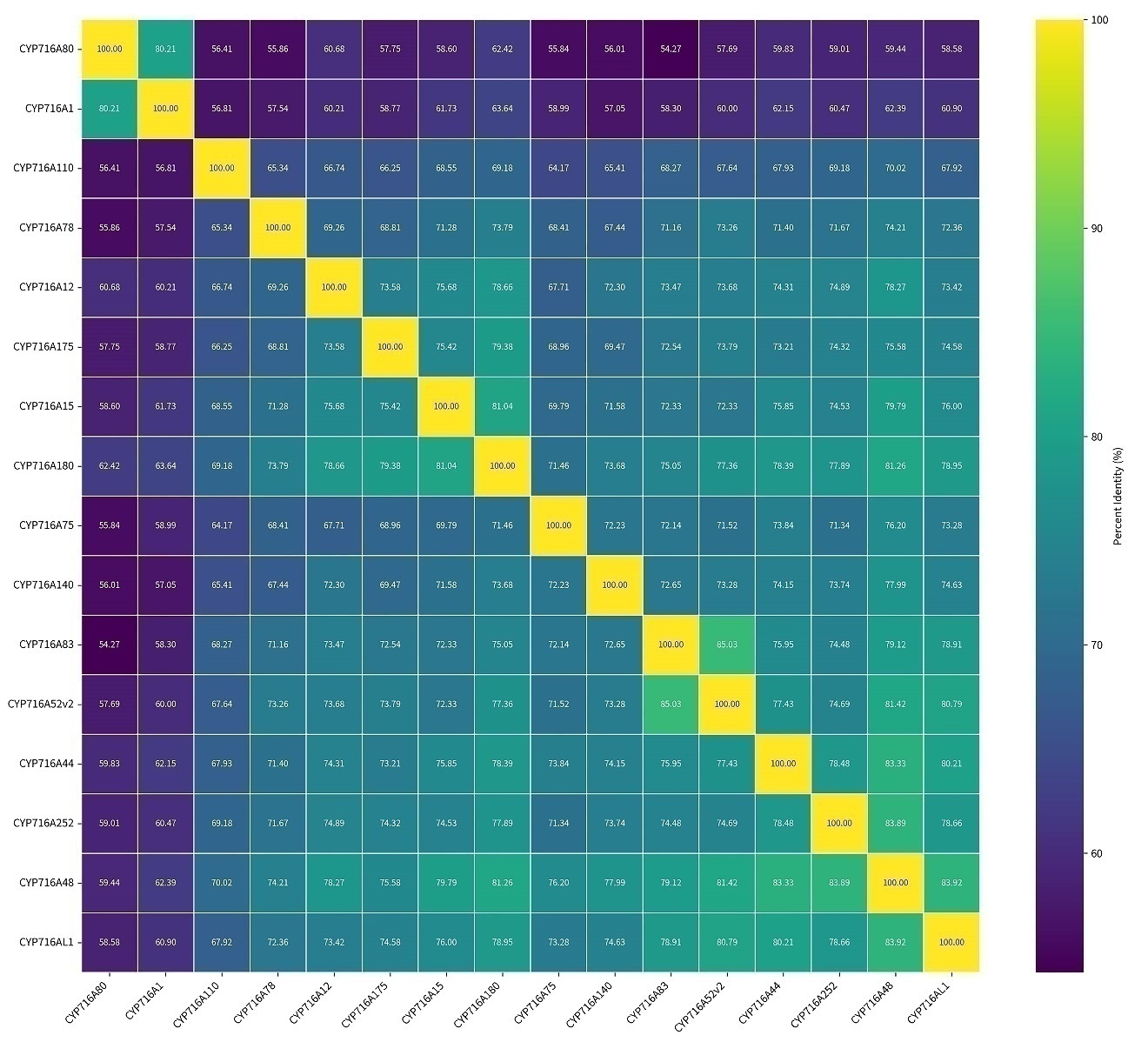


**Figure S9. Heat map of pairwise amino acid sequence identities among 16 CYP716A cytochrome P450 proteins.**

**The heat map was generated from a multiple sequence alignment performed using Clustal2.1 (Clustal Omega). Each cell shows the exact percent identity between protein pairs. Darker purple/blue shades indicate lower sequence identity (minimum 54.27%), whereas brighter yellow/green shades indicate higher sequence identity (maximum 100%). The diagonal represents self-comparisons (100% identity). Protein names are displayed in the same order along both rows and columns as in the original Clustal output.**

**Table S1. CYP716A monooxygenases family members from diverse plant species.**

| **No.** | **CYP name** | **Species** | **Function** | **Accession number** |
| --- | --- | --- | --- | --- |
| 1 | *CYP716A48* | *Olea europaea* | C-28 oxidative | GenBank: AB706294.1 |
| 2 | *CrCYP716AL1* | *Catharanthus roseus* | C-28 oxidative | GenBank: JN565975 |
| 3 | *CYP716A12* | *Medicago truncatula* | C-28 oxidative | GenBank: FN995112 |
| 4 | *CYP716A15* | *Vitis vinifera* | C-28 oxidative | GeneID:100251813 |
| 5 | *CYP716A52v2* | *Panax ginseng* | C-28 oxidative | GenBank: JX036032.1 |
| 6 | *CYP716A44* | *Solanum lycopersicum* | C-28 oxidative | GenBank: AK329870.1 |
| 7 | *CYP716A83* | *Centella asiatica* | C-28 oxidative | GenBank: KU878849 |
| 8 | *CYP716A75* | *Maesa lanceolata* | C-28 oxidative | GenBank: KF318733.1 |
| 9 | *CYP716A110* | *Aquilegia coerulea* | C-28 oxidative | GenBank: KU878864.1 |
| 10 | *CYP716A140* | *Platycodon grandiflorus* | C-28 oxidative | UniProt: A0A1I9Q5Y8 |
| 11 | *CYP716A252* | *Ocimum basilicum* | C-28 oxidative | GenBank: AFZ40057.1 |
| 12 | *CYP716A80* | *Barbarea vulgaris* | C-28 oxidative | GenBank: ALR73782.1 |
| 13 | *CYP716A78* | *Chenopodium quinoa* | C-28 oxidative | GenBank: ANY30853.1 |
| 14 | *CYP716A175* | *Malus x domestica* | C-28 oxidative | GenBank: XP_008391096.1 |
| 15 | *CYP716A180* | *Betula platyphylla* | C-28 oxidative | GenBank: AHL46848.1 |
| 16 | *CYP716A1* | *Arabidopsis thaliana* | C-28 oxidative | GenBank: EFH44718.1 |

**Table S2. Native genes used in this study.**

| **No.** | **Gene name** | **Locus tag** | **Function** |
| --- | --- | --- | --- |
| 1 | *RIB1* | YALI0E02288g | GTP cyclohydrolase II |
| 2 | *RIB2* | YALI0F12199g | Diaminohydroxyphosphoribosylaminopyrimidine deaminase/reductase |
| 3 | *RIB3* | YALI0B08965g | 3,4-dihydroxy-2-butanone-4-phosphate synthase |
| 4 | *FMN1* | YALI0B01826g | Riboflavin kinase converting riboflavin to FMN |
|  | *FAD1* | YALI0D25564 | Flavin adenine dinucleotide synthetase catalyzing the ATP-dependent conversion of FMN to FAD |
| 5 | *HEM2* | YALI0F20790g | Aminolevulinic acid dehydratase |
| 6 | *HEM3* | YALI0F26609g | Porphobilinogen deaminase |
| 7 | *HEM12* | YALI0C01716g | Uroporphyrinogen decarboxylase |
| 8 | *FTR1* | YALI0A04917g | High-affinity iron permease |
| 9 | *FET3* | YALI1A21283g | Multicopper oxidase |
| 10 | *FRE1* | YALI0E12551g | Ferric reductase |
| 11 | *HMX1* | YALI1D33552g | Heme oxygenase |


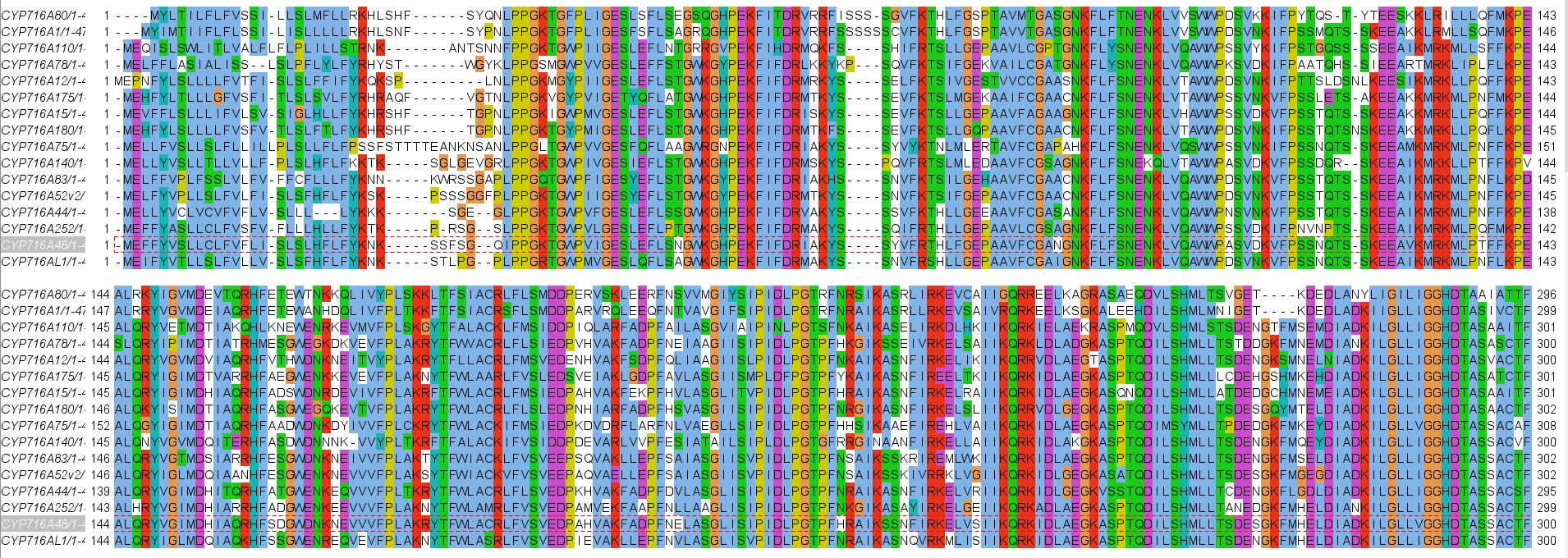


**Figure S10. Multiple sequence alignment of 16 CYP716A cytochrome P450 proteins (residues 1–300) generated using Clustal Omega and visualized with Jalview.**


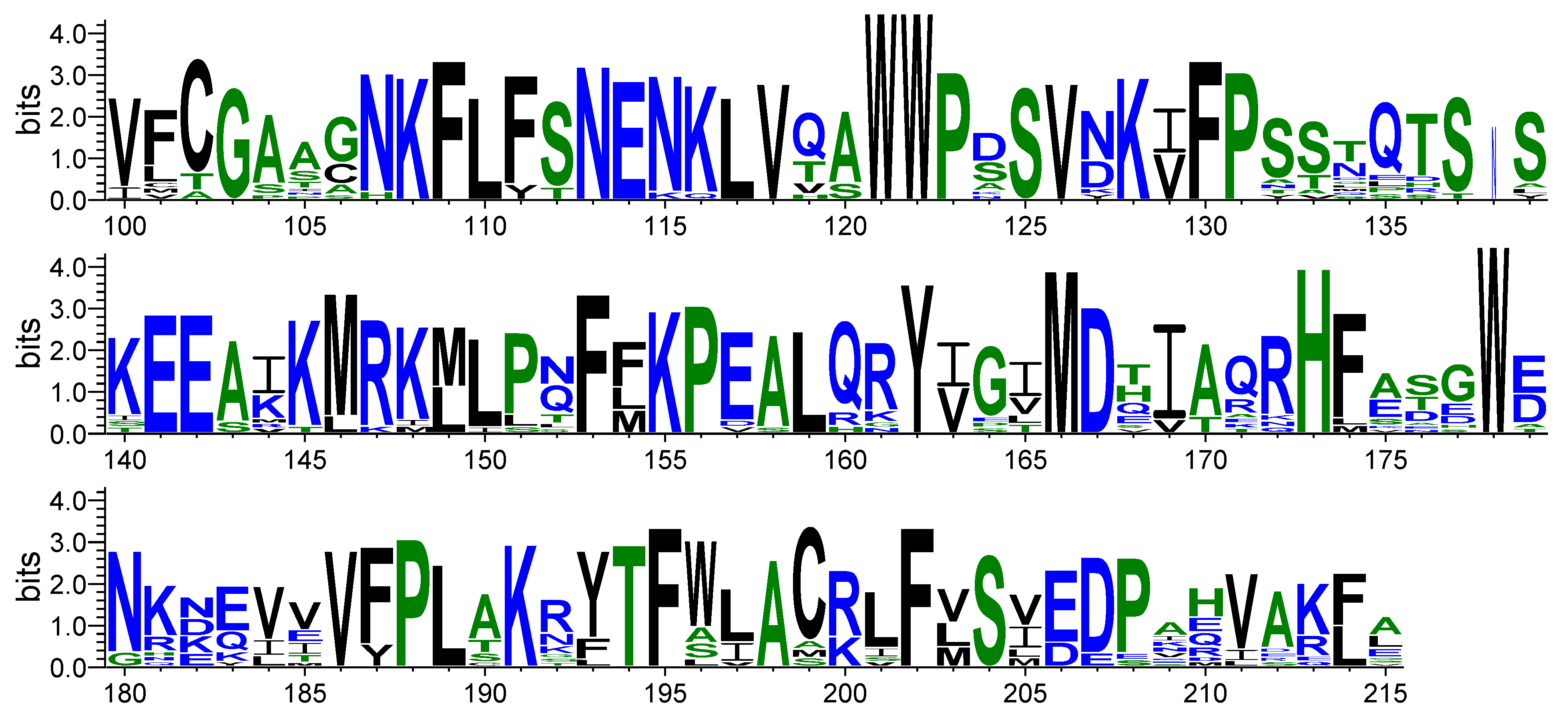
**Figure S11. Multiple sequence alignment of 16 CYP716A cytochrome P450 proteins (residues 100–215) generated using Clustal Omega and and visualized in WebLogo.**

**Table S3: Plasmids used in this study**

| **Plasmids** | **Description** | **Sources** |
| --- | --- | --- |
| pYLT00 | *Amp*,*URA3* marker, TEF promoter and XPR2 terminator | Lab stock |
| pYLT01 | *Amp*, *URA3* marker, TDH promoter and ICLt terminator | Lab stock |
| pYLT02 | pYLT001,2:A2-P_TEF_-*MtCYP716A12*-T_XPR2_-P_TDH_-*AtCPR1*- T_ICLt_ | This study |
| pYLT03 | pYLT001,2:A2-P_TEF_-*CrCYP716AL1*-T_XPR2_-P_TDH_-*AtCPR1*- T_ICLt_ | This study |
| pYLT04 | pYLT001,2:A2-P_TEF_-*CaCYP716A83*-T _XPR2_-P_TDH_-*AtCPR1*- T_ICLt_ | This study |
| pYLT05 | pYLT001,2:A2-P_TEF_-*OeCYP716A48*-T _XPR2_-P_TDH_-*AtCPR1*- T_ICLt_ | This study |
| pYLT06 | pYLT001,2:B1-P_TEF_-*OeCYP716A48*{GGGGS}*AtCPR1*-T_XPR2_ | This study |
| pYLT07 | pYLT001,2:B1-P_TEF_-*OeCYP716A48*{**(**GGGGS)2}*AtCPR1*-T_XPR2_ | This study |
| pYLT08 | pYLT001,2:B1-P_TEF_-*OeCYP716A48*{(GGGGS)3}*AtCPR1*-T_XPR2_ | This study |
| pYLT09 | pYLT001,2:B1-P_TEF_-*OeCYP716A48*{GSG}*AtCPR1*-T_XPR2_ | This study |
| pYLT 10 | pYLT001,2:B1-P_TEF_-*OeCYP716A48*{**(**GSG)2}*AtCPR1*-T_XPR2_ | This study |
| pYLT11 | pYLT001,2:B1-P_TEF_-*OeCYP716A48*{TPTP}*AtCPR1*-T_XPR2_ | This study |
| pYLT12 | pYLT001,2:B1-P_TEF_-*OeCYP716A48*{(TPTP)2}*AtCPR1*-T_XPR2_ | This study |
| pYLT13 | pYLT001,2:B1-P_TEF_-*OeCYP716A48*{(TPTP)3}*AtCPR1*-T_XPR2_ | This study |
| pYLT^D114A^ | pYLT001,2: B1-PTEF-*OeCYP716A48^D114A^*-T XPR2-P TDH-*AtCPR1*- TICLt | This study |
| pYLT^S120A^ | pYLT001,2:B1-P_TEF_-*OeCYP716A48^S120A^*-T _XPR2_-P_TDH_-*AtCPR1*- T_ICLt_ | This study |
| pYLT^T123A^ | pYLT001,2:B1-P_TEF_-*OeCYP716A48*^T123A^-T _XPR2_-P_TDH_-*AtCPR1*- T_ICLt_ | This study |
| pYLT^L211A^ | pYLT001,2:B1-P_TEF_-*OeCYP716A48^L211A^*-T _XPR2_-P_TDH_-*AtCPR1*- T_ICLt_ | This study |
| pYLT^D114C^ | pYLT001,2:B1-P_TEF_-*OeCYP716A48*^D114C^-T _XPR2_-P_TDH_-*AtCPR1*- T_ICLt_ | This study |
| pYLT^D114E^ | pYLT001,2:B1-P_TEF_-*OeCYP716A48*^D114E^-T _XPR2_-P_TDH_-*AtCPR1*- T_ICLt_ | This study |
| pYLT^D114F^ | pYLT001,2:B1-P_TEF_-*OeCYP716A48*^D114F^-T _XPR2_-P_TDH_-*AtCPR1*- T_ICLt_ | This study |
| pYLT^D114G^ | pYLT001,2:B1-P_TEF_-*OeCYP716A48*^D114G^-T _XPR2_-P_TDH_-*AtCPR1*- T_ICLt_ | This study |
| pYLT^D114H^ | pYLT001,2:B1-P_TEF_-*OeCYP716A48*^D114H^-T _XPR2_-P_TDH_-*AtCPR1*- T_ICLt_ | This study |
| pYLT^D114I^ | pYLT001,2:B1-P_TEF_-*OeCYP716A48*^D114I^-T _XPR2_-P_TDH_-*AtCPR1*- T_ICLt_ | This study |
| pYLT^D114K^ | pYLT001,2:B1-P_TEF_-*OeCYP716A48*^D114K^-T _XPR2_-P_TDH_-*AtCPR1*- T_ICLt_ | This study |
| pYLT^D114L^ | pYLT001,2:B1-P_TEF_-*OeCYP716A48*^D114L^-T _XPR2_-P_TDH_-*AtCPR1*- T_ICLt_ | This study |
| pYLT^D114M^ | pYLT001,2:B1-P_TEF_-*OeCYP716A48*^D114M^-T _XPR2_-P_TDH_-*AtCPR1*- T_ICLt_ | This study |
| pYLT^D114N^ | pYLT001,2:B1-P_TEF_-*OeCYP716A48*^D114N^-T _XPR2_-P_TDH_-*AtCPR1*- T_ICLt_ | This study |
| pYLT^D114P^ | pYLT001,2:B1-P_TEF_-*OeCYP716A48*^D114P^-T _XPR2_-P_TDH_-*AtCPR1*- T_ICLt_ | This study |
| pYLT^D114Q^ | pYLT001,2:B1-P_TEF_-*OeCYP716A48*^D114Q^-T _XPR2_-P_TDH_-*AtCPR1*- T_ICLt_ | This study |
| pYLA^D114R^ | pYLT001,2:B1-P_TEF_-*OeCYP716A48*^D114R^-T _XPR2_-P_TDH_-*AtCPR1*- T_ICLt_ | This study |
| pYLT^D114S^ | pYLT001,2:B1-P_TEF_-*OeCYP716A48*^D114S^-T _XPR2_-P_TDH_-*AtCPR1*- T_ICLt_ | This study |
| pYLT^D114T^ | pYLT001,2:B1-P_TEF_-*OeCYP716A48^D114T^*-T _XPR2_-P_TDH_-*AtCPR1*- T_ICLt_ | This study |
| pYLA^D114V^ | pYLT001,2:B1-P_TEF_-*OeCYP716A48*^D114V^-T _XPR2_-P_TDH_-*AtCPR1*- T_ICLt_ | This study |
| pYLT^D114W^ | pYLT001,2:B1-P_TEF_-*OeCYP716A48*^D114W^-T _XPR2_-P_TDH_-*AtCPR1*- T_ICLt_ | This study |
| pYLA^D114Y^ | pYLT001,2:B1-P_TEF_-*OeCYP716A48*^D114Y^-T _XPR2_-P_TDH_-*AtCPR1*- T_ICLt_ | This study |
| pYLT^L211V^ | pYLT001,2:B1-P_TEF_-*OeCYP716A48*^L211V^-T _XPR2_-P_TDH_-*AtCPR1*- T_ICLt_ | This study |
| pYLT^L211I^ | pYLT001,2:B1-P_TEF_-*OeCYP716A48*^L211I^-T _XPR2_-P_TDH_-*AtCPR1*- T_ICLt_ | This study |
| pYLT^L211M^ | pYLT001,2:B1-P_TEF_-*OeCYP716A48*^L211M^-T _XPR2_-P_TDH_-*AtCPR1*- T_ICLt_ | This study |
| pYLT^L211F^ | pYLT001,2:B1-P_TEF_-*OeCYP716A48*^L211F^-T _XPR2_-P_TDH_-*AtCPR1*- T_ICLt_ | This study |
| pYLT^L211W^ | pYLT001,2:B1-P_TEF_-*OeCYP716A48*^L211W^-T _XPR2_-P_TDH_-*AtCPR1*- T_ICLt_ | This study |
| pYLT^L211G^ | pYLT001,2:B1-P_TEF_-*OeCYP716A48*^L211G^-T _XPR2_-P_TDH_-*AtCPR1*- T_ICLt_ | This study |
| pYLT^L211S^ | pYLT001,2:B1-P_TEF_-*OeCYP716A48*^L211S^-T _XPR2_-P_TDH_-*AtCPR1*- T_ICLt_ | This study |
| pYLT^L211T^ | pYLT001,2:B1-P_TEF_-*OeCYP716A48*^L211T-^T _XPR2_-P_TDH_-*AtCPR1*- T_ICLt_ | This study |
| pYLT^L211C^ | pYLT001,2:B1-P_TEF_-*OeCYP716A48*^L211C^-T _XPR2_-P_TDH_-*AtCPR1*- T_ICLt_ | This study |
| pYLT^L211P^ | pYLT001,2:B1-P_TEF_-*OeCYP716A48*^L211P^-T _XPR2_-P_TDH_-*AtCPR1*- T_ICLt_ | This study |
| pYLT^L211N^ | pYLT001,2:B1-P_TEF_-*OeCYP716A48*^L211N^-T _XPR2_-P_TDH_-*AtCPR1*- T_ICLt_ | This study |
| pYLT^L211Q^ | pYLT001,2:B1-P_TEF_-*OeCYP716A48*^L211Q^-T _XPR2_-P_TDH_-*AtCPR1*- T_ICLt_ | This study |
| pYLT^L211Y^ | pYLT001,2:B1-P_TEF_-*OeCYP716A48*^L211Y^-T _XPR2_-P_TDH_-*AtCPR1*- T_ICLt_ | This study |
| pYLT^L211D^ | pYLT001,2:B1-P_TEF_-*OeCYP716A48*^L211D^-T _XPR2_-P_TDH_-*AtCPR1*- T_ICLt_ | This study |
| pYLT^L211E^ | pYLT001,2:B1-P_TEF_-*OeCYP716A48*^L211E^-T _XPR2_-P_TDH_-*AtCPR1*- T_ICLt_ | This study |
| pYLT^L211K^ | pYLT001,2:B1-P_TEF_-*OeCYP716A48*^L211K^-T _XPR2_-P_TDH_-*AtCPR1*- T_ICLt_ | This study |
| pYLT^L211R^ | pYLT001,2:B1-P_TEF_-*OeCYP716A48*^L211R^-T _XPR2_-P_TDH_-*AtCPR1*- T_ICLt_ | This study |
| pYLT^L211H^ | pYLT001,2:B1-P_TEF_-*OeCYP716A48*^L211H^-T _XPR2_-P_TDH_-*AtCPR1*- T_ICLt_ | This study |
| pYLT^D114Q/L211F^ | pYLT001,2:B1-P_TEF_-*OeCYP716A48*^D114Q/L211F^-T _XPR2_-P_TDH_-*AtCPR1*- T_ICLt_ | This study |
| pYLT^D114A/L211A^ | pYLT001,2:B1-P_TEF_-*OeCYP716A48*^D114A/L211A^-T _XPR2_-P_TDH_-*AtCPR1*- T_ICLt_ | This study |
| pYLT^D114N/L211A^ | pYLT001,2:B1-P_TEF_-*OeCYP716A48*^D114N/L211A^-T _XPR2_-P_TDH_-*AtCPR1*- T_ICLt_ | This study |
| pYLT^D114N/L211G^ | pYLT001,2:B1-P_TEF_-*OeCYP716A48*^D114N/L211G^-T _XPR2_-P_TDH_-*AtCPR1*- T_ICLt_ | This study |
| pYLT^D114A/L211G^ | pYLT001,2:B1-P_TEF_-*OeCYP716A48*^D114A/L211G^-T _XPR2_-P_TDH_-*AtCPR1*- T_ICLt_ | This study |
| pYLT^D114V/L211M^ | pYLT001,2:B1-P_TEF_-*OeCYP716A48*^D114V/L211M^-T _XPR2_-P_TDH_-*AtCPR1*- T_ICLt_ | This study |
| pYLT14 | pYLT00, A4-P_TEF_-*RIB1*-T_XPR2_ | This study |
| pYLT15 | pYLT00, E6-P_TEF_-*RIB2*-T _XPR2_ | This study |
| pYLT16 | pYLT00, F7::P_TEF_-*RIB3*-T_XPR2_ | This study |
| pYLT17 | pYLT001, A7::P_TEF_-*FMN1*-T _XPR2_- P_TDH_-*FAD1*- T_ICLt_ | This study |
| pYLT18 | pYLT00, F8::P_TEF_-*HEM2*-T_XPR2_ | This study |
| pYLT19 | pYLT00 B5::P_TEF_-*HEM3*-T_XPR2_ | This study |
| pYLT20 | pYLT00, B7::P_TEF_-*HEM12*-T_XPR2_ | This study |
| pYLT21 | pYLT00, B8::P_TEF_-*FTR1*-T_XPR2_ | This study |
| pYLT22 | pYLT00, F12::P_TEF_-*FET3*-T_XPR2_ | This study |
| pYLT23 | pYLT00, F13::P_TEF_-*FRE1*-T_XPR2_ | This study |
| pYLT24 | rDNA- P_TEF_-*OeCYP716A48*^D114Q/L211F^-T _XPR2_-P_TDH_-*AtCPR1*- T_ICLt_- P_TEF_-*CrMAS* -T _XPR2_ | This study |
|  |  |  |

**Table S4: Primers used in this study**

| **Primers** | **Sequence (5 to 3)** | **Description** |
| --- | --- | --- |
| P1 | CCAGCACTTTTTGCAGTACTAACCGCAGGAGCCCAACTTCTACCTGTCG | For amplification of *MtCYP716A12*sequence |
| P2 | CAGATGCATAGCACGCGTGTAGATACTCAAGCTTTATGGGGGTAGAGTCG |  |
| P3 | CACACAAGACATATCTACAGCAATGGAAATCTTCTACGTTACTCTTTTG | For amplification of *CrCYP716AL1*sequence |
| P4 | AACTCGTTAAATATATTTTGCTAAACAAACTGCTCAGGCGTTGATGTGGG |  |
| P5 | AGCACTTTTTGCAGTACTAACCGCAGGAGCTGTTTTTTGTGCCTCTGTTT | For amplification of *CaCYP716A83*sequence |
| P6 | CTCAGATGCATAGCACGCGTGTAGATACTCAGGCCTTGTGGGGAAACA |  |
| P7 | CTTTTTGCAGTACTAACCGCAGGAGTTTTTCTACGTGAGTCTTCTGTG | For amplification of *OeCYP716A48*sequence |
| P8 | CATAGCACGCGTGTAGATACTCATGCGTTCAGGGGATATAGAC |  |
| P9 | CTGAACGCAGGCGGTGGCGGTAGCACCTCCGCCCTGTACGCC | For add GGGGS linker |
| P10 | GGCGGAGGTGCTACCGCCACCGCCTGCGTTCAGGGGATATAGACGAATAG |  |
| P11 | CTGAACGCAGGCGGTGGCGGTAGCGGCGGTGGCGGTAGCACCTCCGCCCTGTACGCC | For add (GGGGS)2 linker |
| P12 | GGCGGAGGTGCTACCGCCACCGCCTGCGTTCAGGGGATATAGACGAATAG |  |
| P13 | CTGAACGCAGGCGGTGGCGGTAGCGGCGGTGGCGGTAGCGGCGGTGGCGGTAGCACCTCCGCCCTGTACGCC | For add (GGGGS)3 linker |
| P14 | GGCGGAGGTGCTACCGCCACCGCCGCTACCGCCACCGCCGCTACCGCCACCGCCTGCGTTCAGGGGATATAGACGAATAG |  |
| P15 | CTGAACGCAGGCAGCGGCACCTCCGCCCTGTACGCC | For add GSG linker |
| P16 | GGCGGAGGTGCCGCTGCCTGCGTTCAGGGGATATAGACGAATAG |  |
| P17 | CTGAACGCAGGCAGCGGCGGCAGCGGCACCTCCGCCCTGTACGCC | For add (GSG)2 linker |
| P18 | GGCGGAGGTGCCGCTGCCGCCGCTGCCTGCGTTCAGGGGATATAGACGAATAG |  |
| P19 | CTGAACGCAACCCCCACCCCCACCTCCGCCCTGTACGC | For add TPTP linker |
| P20 | GGCGGAGGTGGGGGTGGGGGTTGCGTTCAGGGGATATAGACGAATAG |  |
| P21 | CTGAACGCAACCCCCACCCCCACCCCCACCCCCACCTCCGCCCTGTACGCCTC | For add (TPTP)2 linker |
| P22 | GGCGGAGGTGGGGGTGGGGGTGGGGGTGGGGGTTGCGTTCAGGGGATATAGACGAATAG |  |
| P23 | CTGAACGCAACCCCCACCCCCACCCCCACCCCCACCCCCACCCCCACCCCCACCCCCACCCCCACCTCCGCCCTGTACGC | For add (TPTP)3 linker |
| P24 | GGCGGAGGTGGGGGTGGGGGTGGGGGTGGGGGTGGGGGTGGGGGTGGGGGTGGGGGTGGGGGTTGCGTTCAGGGGATATAGACGAATAG |  |
| P25 | GCTTCCGTTGCGAAGGTTTTCCCCTCCAGCAAC | For add D114A mutation |
| P26 | GAAAACCTTCGCAACGGAAGCAGGCCACCAG |  |
| P27 | TTCCCCTCCGCGAACCAGACTTCTTCTAAGGAGGAGGC | For add S120A mutation |
| P28 | AGTCTGGTTCGCGGAGGGGAAAACCTTGTCAACG |  |
| P29 | AGCAACCAGGCGTCTTCTAAGGAGGAGGCGGTGAAG | For add T123A mutation |
| P30 | CTTAGAAGACGCCTGGTTGCTGGAGGGGAAAAC |  |
| P31 | GCATCTGGCGCCATTAGCATTCCTATTGATCTGCCTGGTAC | For add L211A mutation |
| P32 | AATGCTAATGGCGCCAGATGCTAGCTCGTTGAATG |  |
| P33 | GCTTCCGTTTGGAAGGTTTTCCCCTCCAGCAAC | For add D114W mutation |
| P34 | GAAAACCTTCCAAACGGAAGCAGGCCACCAG |  |
| P35 | GCTTCCGTTAACAAGGTTTTCCCCTCCAGCAAC | For add D114N mutation |
| P36 | GAAAACCTTGTTAACGGAAGCAGGCCACCAG |  |
| P37 | GCTTCCGTTAGCAAGGTTTTCCCCTCCAGCAAC | For add D114Smutation |
| P38 | GAAAACCTTGCTAACGGAAGCAGGCCACCAG |  |
| P39 | GCTTCCGTTACTAAGGTTTTCCCCTCCAGCAAC | For add D114Tmutation |
| P40 | GAAAACCTTAGTAACGGAAGCAGGCCACCAG |  |
| P41 | GCTTCCGTTTGTAAGGTTTTCCCCTCCAGCAAC | For add D114Cmutation |
| P42 | GAAAACCTTACAAACGGAAGCAGGCCACCAG |  |
| P43 | GCTTCCGTTGGCAAGGTTTTCCCCTCCAGCAAC | For add D114Gmutation |
| P44 | GAAAACCTTGCCAACGGAAGCAGGCCACCAG |  |
| P45 | GCTTCCGTTCCCAAGGTTTTCCCCTCCAGCAAC | For add D114P mutation |
| P46 | GAAAACCTTGGGAACGGAAGCAGGCCACCAG |  |
| P47 | GCTTCCGTTGTTAAGGTTTTCCCCTCCAGCAAC | For add D114V mutation |
| P48 | GAAAACCTTAACAACGGAAGCAGGCCACCAG |  |
| P49 | GCTTCCGTTTTGAAGGTTTTCCCCTCCAGCAAC | For add D114L mutation |
| P50 | GAAAACCTTCAAAACGGAAGCAGGCCACCAG |  |
| P51 | GCTTCCGTTATCAAGGTTTTCCCCTCCAGCAAC | For add D114I mutation |
| P52 | GAAAACCTTGATAACGGAAGCAGGCCACCAG |  |
| P53 | GCTTCCGTTATGAAGGTTTTCCCCTCCAGCAAC | For add D114M mutation |
| P54 | GAAAACCTTCATAACGGAAGCAGGCCACCAG |  |
| P55 | GCTTCCGTTTTTAAGGTTTTCCCCTCCAGCAAC | For add D114F mutation |
| P56 | GAAAACCTTAAAAACGGAAGCAGGCCACCAG |  |
| P57 | GCTTCCGTTTACAAGGTTTTCCCCTCCAGCAAC | For add D114Y mutation |
| P58 | GAAAACCTTGTAAACGGAAGCAGGCCACCAG |  |
| P59 | GCTTCCGTTCAAAAGGTTTTCCCCTCCAGCAAC | For add D114Q mutation |
| P60 | GAAAACCTTTTGAACGGAAGCAGGCCACCAG |  |
| P61 | GCTTCCGTTCACAAGGTTTTCCCCTCCAGCAAC | For add D114H mutation |
| P62 | GAAAACCTTGTGAACGGAAGCAGGCCACCAG |  |
| P63 | GCTTCCGTTAAAAAGGTTTTCCCCTCCAGCAAC | For add D114K mutation |
| P64 | GAAAACCTTTTTAACGGAAGCAGGCCACCAG |  |
| P65 | GCTTCCGTTAGGAAGGTTTTCCCCTCCAGCAAC | For add D114R mutation |
| P66 | GAAAACCTTCCTAACGGAAGCAGGCCACCAG |  |
| P67 | GCTTCCGTTGAGAAGGTTTTCCCCTCCAGCAAC | For add D114E mutation |
| P68 | GAAAACCTTCTCAACGGAAGCAGGCCACCAG |  |
| P69 | GCATCTGGCTTCATTAGCATTCCTATTGATCTGCCTGGTAC | For add L211F mutation |
| P70 | AATGCTAATGAAGCCAGATGCTAGCTCGTTGAATG |  |
| P71 | GCATCTGGCGTCATTAGCATTCCTATTGATCTGCCTGGTAC | For add L211V mutation |
| P72 | CTTGGAAAACATTCCATCACAGACGACGGTGAG |  |
| P73 | GCATCTGGCATTATTAGCATTCCTATTGATCTGCCTGGTAC | For add L211I mutation |
| P74 | AATGCTAATAATGCCAGATGCTAGCTCGTTGAATG |  |
| P75 | GCATCTGGCCCCATTAGCATTCCTATTGATCTGCCTGGTAC | For add L211P mutation |
| P76 | AATGCTAATGGGGCCAGATGCTAGCTCGTTGAATG |  |
| P77 | GCATCTGGCTCCATTAGCATTCCTATTGATCTGCCTGGTAC | For add L211S mutation |
| P78 | AATGCTAATGGAGCCAGATGCTAGCTCGTTGAATG |  |
| P79 | GCATCTGGCACTATTAGCATTCCTATTGATCTGCCTGGTAC | For add L211T mutation |
| P80 | AATGCTAATAGTGCCAGATGCTAGCTCGTTGAATG |  |
| P81 | GCATCTGGCTGCATTAGCATTCCTATTGATCTGCCTGGTAC | For add L211C mutation |
| P82 | AATGCTAATGCAGCCAGATGCTAGCTCGTTGAATG |  |
| P83 | GCATCTGGCATGATTAGCATTCCTATTGATCTGCCTGGTAC | For add L211M mutation |
| P84 | AATGCTAATCATGCCAGATGCTAGCTCGTTGAATG |  |
| P85 | GCATCTGGCAATATTAGCATTCCTATTGATCTGCCTGGTAC | For add L211N mutation |
| P86 | AATGCTAATATTGCCAGATGCTAGCTCGTTGAATG |  |
| P87 | GCATCTGGCCAAATTAGCATTCCTATTGATCTGCCTGGTAC | For add L211Q mutation |
| P88 | AATGCTAATTTGGCCAGATGCTAGCTCGTTGAATG |  |
| P89 | GCATCTGGCGATATTAGCATTCCTATTGATCTGCCTGGTAC | For add L211D mutation |
| P90 | AATGCTAATATCGCCAGATGCTAGCTCGTTGAATG |  |
| P91 | GCATCTGGCGAGATTAGCATTCCTATTGATCTGCCTGGTAC | For add L211E mutation |
| P92 | AATGCTAATCTCGCCAGATGCTAGCTCGTTGAATG |  |
| P93 | GCATCTGGCAAGATTAGCATTCCTATTGATCTGCCTGGTAC | For add L211K mutation |
| P94 | AATGCTAATCTTGCCAGATGCTAGCTCGTTGAATG |  |
| P95 | GCATCTGGCCGAATTAGCATTCCTATTGATCTGCCTGGTAC | For add L211R mutation |
| P96 | AATGCTAATTCGGCCAGATGCTAGCTCGTTGAATG |  |
| P97 | GCATCTGGCCACATTAGCATTCCTATTGATCTGCCTGGTAC | For add L211H mutation |
| P98 | AATGCTAATGTGGCCAGATGCTAGCTCGTTGAATG |  |
| P99 | GCATCTGGCGGTATTAGCATTCCTATTGATCTGCCTGGTAC | For add L211G mutation |
| P100 | AATGCTAATACCGCCAGATGCTAGCTCGTTGAATG |  |
| P101 | GCATCTGGCTACATTAGCATTCCTATTGATCTGCCTGGTAC | For add L211Y mutation |
| P102 | AATGCTAATGTAGCCAGATGCTAGCTCGTTGAATG |  |
| P103 | GCATCTGGCTGGATTAGCATTCCTATTGATCTGCCTGGTAC | For add L211W mutation |
| P104 | AATGCTAATCCAGCCAGATGCTAGCTCGTTGAATG |  |
| P105 | CTTTTTGCAGTACTAACCGCAGATGAGCAAGCCTATTGATATTCCCGATAC | For amplification of *RIB1*sequence |
| P106 | CATAGCACGCGTGTAGATACTTATTTTGCCATGGGTTCGCTGAG |  |
| P107 | CTTTTTGCAGTACTAACCGCAGATGCTACACGAGCTGGTGAAAATTC | For amplification of *RIB2*sequence |
| P108 | CATAGCACGCGTGTAGATACCTAGTTCTTTGGATGGCCCTTCTTTGC |  |
| P109 | CTTTTTGCAGTACTAACCGCAGATGAGCCAGTTTTCCGCCATTC | For amplification of *RIB3*sequence |
| P110 | CATAGCACGCGTGTAGATACTTACTGCTGCTCGATGTACTTCACC |  |
| P111 | CTTTTTGCAGTACTAACCGCAGATGATGATTAGTTGCACCAGAACCG | For amplification of *FMN1*sequence |
| P112 | CATAGCACGCGTGTAGATACTCACTGCCAGAACTGGTCCTTC |  |
| P113 | CTTTTTGCAGTACTAACCGCAGATGGTCCACAAAGCCGAGTACC | For amplification of *HEM2*sequence |
| P114 | CATAGCACGCGTGTAGATACTTAGTGGTGGTGGTCCAACCAC |  |
| P115 | CTTTTTGCAGTACTAACCGCAGATGTCAGTAGAGGAACGACCAGTG | For amplification of *HEM3*sequence |
| P116 | CATAGCACGCGTGTAGATACCTATTTAATATTTTCCAGGTGAATGGCGTCCAG |  |
| P117 | CTTTTTGCAGTACTAACCGCAGATGCACATGGCTCACAATCGAAC | For amplification of *HEM12* sequence |
| P118 | CATAGCACGCGTGTAGATACTTAGACCCACTCACGGGGCTC |  |
| P119 | CTTTTTGCAGTACTAACCGCAGATGGTCAACTACGCGGACTTTTTC | For amplification of *FTR1*sequence |
| P120 | CATAGCACGCGTGTAGATACTTAAGAAGTGCTGGCAAGAAGAGGAG |  |
| P121 | CTTTTTGCAGTACTAACCGCAGATGTCATACAAGAAGATTGGCGGC | For amplification of *FET3* sequence |
| P122 | CATAGCACGCGTGTAGATACTTAGTTAGTTTGTCCCTCCTTTTGGCC |  |
| P123 | CTTTTTGCAGTACTAACCGCAGATGCCACGCATTCTGGCAAG | For amplification of *FRE1* sequence |
| P124 | CATAGCACGCGTGTAGATACTTACCATCCAAACCGCTCGG |  |
| P125 | GAAGGAAAGGTGAAAAGAACTTTGGAGTTTGGCGCCCGTTTTTTC | For amplification of *rDNA* sequence |
| P126 | CGTCAGAACCGCTACGGGACACGGGCATCTCACTTGC |  |

**Table S5. Main strains used in this study.**

| **Strains** | **Description** | **Source** |
| --- | --- | --- |
| *E. coli* | JM109 | Lab stock |
| Pof1 | ATCC MYA-2613, *ura3-302, leu2-270, xpr2-322, axp-2* | [1] |
|  |  |  |
| YU-0 | Pof1, E4::*P_TEF_- CrMAS*^L323A^ *-T_XPR2_*, *ERG1*::P_TEF_-*ERG1*^T202V/M308L^-T_XPR2_, A1::*P_TEF_-ERG1*^T202V/M308L^ *-T_XPR2_,* E2::P_TEF_-*CrMAS*^L323A^-T_XPR2_, F1::P_TEF_-*CrMAS*^L323A^-T_XPR2_, B4::P_TEF_-*CrMAS*^L323A^-T_XPR2_, E5::P_TEF_-*CrMAS*^L323A^-T_XPR2_, B3::P_TEF_-*ZWF1*-T_XPR2_, P_TDH_-*ZWF1*-T_ICLt_, F4::P_TEF_-*GND1*-T_XPR2_ | Lab stock |
| YU-1 | YU-0, A2::P_TEF_-*MtCYP716A12*-T_XPR2_-P_TDH_-*AtCPR1*- T_ICLt_ | This study |
| YU-2 | YU-0, A2::P_TEF_-*CrCYP716AL1*-T_XPR2_-P_TDH_-*AtCPR1*- T_ICLt_ | This study |
| YU-3 | YU-0, A2::P_TEF_-*CaCYP716A83*-T _XPR2_-P_TDH_-*AtCPR1*- T_ICLt_ | This study |
| YU-4 | YU-0, A2::P_TEF_-*OeCYP716A48*-T _XPR2_-P_TDH_-*AtCPR1*- T_ICLt_ | This study |
| YU-5 | YU-0, B1::P_TEF_-*OeCYP716A48*{GGGGS}*AtCPR1*-T_XPR2_ | This study |
| YU-6 | YU-0, B1::P_TEF_-*OeCYP716A48*{**(**GGGGS)2}*AtCPR1*-T_XPR2_ | This study |
| YU-7 | YU-0, B1::P_TEF_-*OeCYP716A48*{(GGGGS)3}*AtCPR1*-T_XPR2_ | This study |
| YU-8 | YU-0 B1::P_TEF_-*OeCYP716A48*{GSG}*AtCPR1*-T_XPR2_ | This study |
| YU-9 | YU-0, : B1::P_TEF_-*OeCYP716A48*{**(**GSG)2}*AtCPR1*-T_XPR2_ | This study |
| YU-10 | YU-0, B1::P_TEF_-*OeCYP716A48*{TPTP}*AtCPR1*-T_XPR2_ | This study |
| YU-11 | YU-0, B1::P_TEF_-*OeCYP716A48*{(TPTP)2}*AtCPR1*-T_XPR2_ | This study |
| YU-12 | YU-0 B1::P_TEF_-*OeCYP716A48*{(TPTP)3}*AtCPR1*-T_XPR2_ | This study |
| YU-13 | YU-0, B1::P_TEF_-*OeCYP716A48*^D114Q/L211F^-T _XPR2_-P_TDH_-*AtCPR1*- T_ICLt_ | This study |
| YU-14 | YU-13, A4::P_TEF_-*RIB1*-T_XPR2_ | This study |
| YU-15 | YU-14, E6::P_TEF_-*RIB2*-T _XPR2_ | This study |
| YU-16 | YU-15, F7::P_TEF_-*RIB3*-T_XPR2_ | This study |
| YU-17 | YU-16, A7::P_TEF_-*FMN1*-T _XPR2_- P_TDH_-*FAD1*- T_ICLt_ | This study |
| YU-18 | YU-17, F8::P_TEF_-*HEM2*-T_XPR2_ | This study |
| YU-19 | YU-18, B5::P_TEF_-*HEM3*-T_XPR2_ | This study |
| YU-20 | YU-19, B7::P_TEF_-*HEM12*-T_XPR2_ | This study |
| YU-21 | YU-20,*ΔHMX1* | This study |
| YU-22 | YU-21, B8::P_TEF_-*FTR1*-T_XPR2_ | This study |
| YU-23 | YU-22, F12::P_TEF_-*FET3*-T_XPR2_ | This study |
| YU-24 | YU-23, F13::P_TEF_-*FRE1*-T_XPR2_ | This study |
| YU-25 | YU-24, rDNA- P_TEF_-*OeCYP716A48*^D114Q/L211F^-T _XPR2_-P_TDH_-*AtCPR1*- T_ICLt_-P_TEF_-*CrMAS* -T _XPR2_ | This study |

**Table S6. Comparative UA production and α-amyrin accumulation in engineered Y. lipolytica strains under shake flask batch culture conditions.**

| **Strain** | **Genetic Modification** | **UA Production** | **α-Amyrin Production** |
| --- | --- | --- | --- |
| Strain YU-0 | laboratory stock strain | Not detectable | 197 ± 6 mg/L |
| Strain YU-4 | Single-copy *OeCYP716A48* + *AtCPR1* | 13 ± 1 mg/L | 175 ± 7 mg/L |
| Strain YU-13 | Single-copy *OeCYP716A48^D114Q^*^/L211F^ + *AtCPR1* | 66 ± 3 mg/L | 109 ± 3 mg/L |
| Strain YU-17 | Single-copy *OeCYP716A48^D114Q^*^/L211F^ + *AtCPR1+* Engineering FAD Biosynthesis | 110 ± 4 mg/L | 85 ± 3 mg/L |
| Strain YU-21 | Single-copy *OeCYP716A48^D114Q^*^/L211F^ + *AtCPR1+* Engineering FAD Biosynthesis + Optimization of Heme Supply | 153 ± 5 mg/L | 42 ± 1 mg/L |
| Strain YU-24 | Single-copy *OeCYP716A48^D114Q^*^/L211F^ + *AtCPR1+* Engineering FAD Biosynthesis + Optimization of Heme Supply + Improve iron uptake system | 187 ± 6 mg/L | 9.0 ± 0.3 mg/L |
| Strain YU-25 | Single-copy *OeCYP716A48^D114Q^*^/L211F^ + *AtCPR1+* Engineering FAD Biosynthesis + Optimization of Heme Supply + Improve iron uptake system + Multicopy *CrMAS*, *OeCYP716A48*^D114Q/L211F^, and *AtCPR1* (rDNA loci) | 205 ± 7 mg/L | Not detectable |
| Strain YU-25 | Fed-batch fermentation in 5-L bioreactor | 813 ± 24 mg/L | Not detectable |

**Table S7. Codon-optimized nucleotide sequence of the heterologous gene used in this study.**

***CrMAS (Catharanthus rosеus)* Sequence (5'-3')**

ATGTGGAAGCTCAAGATTGCCAAGGGTAAGGGGCCTTACCTATACAGCACCAACAACTTCGTGGGTCGACAAATTTGGGAATACGATCCCAACGCAGGAACTCCCCAAGAGCGAGAGGCCTTTGAGAAGGCCCGCGAACAGTTCCGAAACAACAGAAAGAAGGGGGTGCACAATCCCTGTGCAGATCTGTTCATGAGAATGCAGCTGATAAAAGAGAACGGTATCGACCTAATGTCCATTCCGCCTGTGCGAGTCGAGGAGAAGGAGGAGCTCACGTTCGAGAAGACCACCATTGCTGTCAAGAAGGCCCTCCGGCTCAACCGTGCCATTCAGGCCACGGACGGCCACTGGCCTGCTGAGAATGCTGGTCCAATGTTCTTCACTCCTCCACTTCTCATTGCGCTGTACATTAGTGGAGCCATCAACACCATTCTGACTTCGGAACACAAGAAAGAGCTGGTCCGCTACATCTACAACCACCAGAACGAGGATGGCGGCTGGGGTTTTTACATTGAGGGACATTCTACTATGATTGGCAGCGCGCTGAGTTACGTGGCTCTCCGGCTATTAGGCGAAGGTCCCGATGATGGAGATGGTGCTGTAGGTAGAGGACGTCAGTGGATCCTCGACCACGGAGGAGCCACGGGTATCCCCTCTTGGGGTAAGACCTACCTTTCTGTTCTCGGTGTCTACGACTGGGATGGATGTAACCCGCTGCCGCCCGAATTCTGGCTCTTTCCTTCGTTCTTCCCTTATCATCCCGCTAAGATGTGGTGCTACTGCCGGACAACATACATGCCGATGTCTTACCTGTACGGAAAGAAGTATCACGGACCTCTGACCCATCTCGTTATGCAACTCAGGCAGGAGATCCACGTGAAGCCCTATGATCAGATTGACTGGAACAAGGCACGACACGATTGCTGCAAGGACGATCTGTACTACCCCCATTCATTCATCCAGGATGCCCTGTGGGACACTCTTAACTACTTTTCAGAGCCCGTCATGAGACGATGGCCCTGTAACAAGATTAGAGAAAAGGCCATGCGAAAGTGCATCAAATATATGCGATACGGCGCCGAGGAGTCGCGCTATATCACCATTGGCTGTGTGGAGAAATCGCTGCAAATGATGTGCTGGTGTGCCCACGACCCGAACTGCGACGAGTTCAAATACCACCTTGCACGTGTGCCTGACTACCTCTGGCTGGCAGAAGATGGCATGAAGATGCAGAGCTTTGGATCTCAGCTCTGGGACTGTACCCTAGCTACCCAGGCTATCATAGCAACCGGAATGGTCGAGGAATATGGAGACACAATCAAAAAGGCGCACTTCTACATCAAGGAGAGCCAGGTGAAGGAGAACCCAAAGGAGGACTTCAAGGCCATGTACCGACATTTCACTAAGGGGTCCTGGACATTTTCCGACCAGGACCAGGGCTGGGTGGTCTCGGACTGCACCGCCGAAGCTCTGAAGTGTCTTCTCGTCTGTTCTCAAATGCCACAAGACCTTGCTGGTGAAAAAGCGGACGTGGAGCGGTTATACGACGCCGTCAACGTCCTTTTGTATCTTCAGTCTCCCGAGTCTGGAGGATTTGCTATCTGGGAGCCACCTGTTCCTCAGCCCTATCTGCAAGTTCTGAACCCCTCTGAATTGTTTGCCGACATCGTTGTCGAACAGGAGCATGTTGAAAATACTGCCTCCATCGTTCAGGCGCTTGTACTGTTTAAACGATTGCACCCCGGCCATCGGGAGAAGGAGATTGAAGTGTCGGTTTCCAAGGCTGTGCGTTTTCTGGAGGGCCGACAGTGGCCCGATGGATCCTGGTACGGGTACTGGGGCATCTGCTTCCTGTACGGCACCATGTTTGTGCTCGGTGGGCTGACTGCGGCTGGCAAGACTTACAAGAACTCCGAGGCTATTCGAAAAGCCGTCAAGTTCTATTTATCAACGCAAAATGAGGAGGGAGGATGGGGAGAGTGTCTGGAATCGTGTCCCTCCATGAAGTACATTCCTCTTGAGGGCAATCGAACCAATCTGGTGCAGACATCCTGGGCTATGCTAGGCCTGATGTACGGTGGCCAGGCAGAACGTGACCCCACCCCTCTCCACAAAGCTGCCAAGCTGCTGATCAACGCCCAGATGGACGACGGCGATTTTCCTCAGCAGGAGATCACTGGTGTATACATGAAGAACTGCATGTTGCATTACGCCGAGTACCGGAACATTTTCCCCCTGTGGGCCTTGGCGGAGTACCGCAAGCGAGTGTGGCCTACGAAAGCTTTGTGA

***AtCPR1 (Arabidopsis thaliana)* Sequence (5'-3')**

ATGACCTCCGCCCTGTACGCCTCTGACCTGTTCAAGCAGCTGAAGTCTATCATGGGCACCGACTCTCTGTCTGACGACGTGGTGCTGGTGATCGCCACCACCTCTCTGGCCCTGGTGGCCGGATTCGTGGTGCTGCTGTGGAAAAAGACCACCGCCGACCGATCTGGCGAGCTGAAGCCCCTGATGATCCCCAAGTCTCTGATGGCCAAGGACGAGGACGATGACCTGGACCTGGGCTCTGGCAAGACCCGAGTGTCTATCTTCTTCGGCACTCAGACCGGCACCGCCGAGGGCTTCGCCAAGGCCCTGTCTGAGGAGATCAAGGCCCGATACGAAAAGGCCGCCGTCAAGGTGATCGACCTGGACGACTACGCCGCCGACGATGATCAGTACGAGGAGAAACTGAAAAAAGAAACCCTGGCTTTCTTCTGTGTGGCTACCTACGGCGACGGCGAGCCCACCGACAACGCCGCCCGATTCTACAAGTGGTTCACCGAGGAGAACGAGCGAGACATCAAGCTGCAGCAGCTGGCCTACGGCGTGTTCGCCCTGGGCAACCGACAGTACGAGCACTTCAACAAGATCGGCATCGTGCTGGACGAGGAGCTGTGTAAGAAGGGCGCCAAGCGACTGATCGAGGTGGGCCTGGGCGACGATGATCAGTCTATCGAGGACGACTTCAACGCCTGGAAGGAGTCTCTGTGGTCTGAGCTGGACAAGCTGCTGAAGGACGAGGATGATAAGTCTGTGGCCACCCCCTACACCGCCGTGATCCCCGAGTACCGAGTGGTGACCCACGACCCCCGATTCACCACTCAGAAGTCTATGGAGTCTAACGTGGCCAACGGCAACACCACCATCGACATCCACCACCCCTGTCGAGTGGACGTGGCCGTGCAGAAGGAGCTGCACACCCACGAGTCTGACCGATCTTGTATCCACCTGGAGTTCGACATCTCTCGAACCGGCATCACCTACGAAACCGGCGACCACGTGGGCGTGTACGCCGAGAACCACGTGGAGATCGTGGAGGAGGCCGGCAAGCTGCTGGGCCACTCTCTGGACCTGGTGTTCTCTATCCACGCCGACAAGGAGGATGGCTCTCCCCTGGAATCTGCCGTGCCCCCTCCCTTCCCCGGACCCTGTACCCTGGGCACTGGACTCGCCCGATATGCCGACCTGCTGAACCCCCCCCGAAAGTCTGCCCTGGTGGCCCTCGCCGCTTACGCCACCGAGCCCTCTGAGGCCGAGAAGCTGAAACACCTGACCTCTCCCGACGGCAAGGACGAGTACTCTCAGTGGATCGTGGCCTCTCAGCGATCTCTGCTGGAGGTGATGGCCGCTTTCCCCTCTGCCAAGCCCCCCCTGGGCGTGTTCTTCGCCGCCATCGCCCCCCGACTGCAGCCCCGATACTACTCTATCTCTTCCTCTCCCCGACTGGCCCCCTCTCGAGTGCACGTGACCTCTGCCCTGGTGTACGGCCCCACCCCCACCGGCCGAATCCACAAGGGCGTGTGTTCTACCTGGATGAAGAACGCCGTGCCCGCCGAGAAGTCTCACGAGTGTTCTGGCGCCCCCATCTTCATCCGAGCCTCTAACTTCAAGCTGCCCTCTAACCCCTCTACCCCCATTGTGATGGTGGGCCCCGGCACCGGCCTGGCCCCTTTCCGAGGCTTCCTGCAAGAGCGAATGGCCCTGAAGGAAGATGGAGAGGAGCTGGGCTCTTCTCTGCTGTTCTTCGGCTGTCGAAACCGACAGATGGACTTCATCTACGAGGACGAGCTGAACAACTTCGTGGACCAAGGCGTGATCTCTGAGCTGATCATGGCCTTCTCTCGAGAGGGCGCTCAGAAGGAGTACGTGCAGCACAAGATGATGGAGAAGGCCGCCCAAGTGTGGGACCTGATCAAGGAGGAGGGCTACCTGTACGTGTGTGGCGACGCCAAGGGCATGGCCCGAGACGTGCACCGAACCCTGCACACCATCGTGCAAGAGCAAGAGGGAGTGTCCTCTTCCGAGGCCGAGGCCATCGTGAAGAAGCTGCAGACCGAGGGCCGATACCTGCGAGACGTGTGGTAA

***MtCYP716A12 (Medicago truncatula)* Sequence (5'-3')**

ATGGAGCCCAACTTCTACCTGTCGTTATTGCTACTTTTTGTGTCCTTCATTTCTCTGAGTCTGTTTTTCATCTTTTACAAGCAGAAGTCGCCACTCAATTTGCCGCCGGGTAAAATGGGATACCCTATTATTGGAGAATCACTCGAGTTCCTGTCAACGGGCTGGAAGGGCCACCCCGAAAAGTTTATTTTTGATAGAATGCGAAAATATTCGTCTGAACTGTTCAAGACCTCCATTGTTGGTGAATCCACTGTGGTATGCTGTGGAGCTGCTAGCAATAAGTTCCTATTTAGCAACGAGAATAAGCTCGTGACCGCCTGGTGGCCCGACTCTGTCAACAAAATTTTCCCAACGACTTCCCTGGACTCCAATCTAAAGGAGGAGTCTATCAAGATGCGTAAACTCTTGCCTCAGTTCTTCAAGCCCGAGGCCCTGCAACGATACGTTGGGGTCATGGATGTGATTGCCCAGCGGCATTTCGTGACCCACTGGGACAACAAGAATGAGATCACAGTTTATCCCCTGGCGAAGCGGTACACTTTCCTTCTTGCTTGCCGACTGTTCATGTCCGTTGAGGACGAAAACCACGTGGCCAAATTTTCCGACCCTTTCCAGCTGATCGCGGCTGGTATCATCTCTCTCCCTATAGACTTGCCTGGAACCCCCTTCAACAAGGCCATCAAGGCTTCCAACTTTATCAGGAAGGAACTCATTAAGATCATCAAACAGCGACGAATCGATCTGGCGGAAGGAACTGCTTCTCCCACCCAGGACATTCTTTCTCACATGCTCCTGACAAGCGACGAGAACGGAAAGTCCATGAACGAGTTGAACATTGCTGATAAGATATTGGGCCTTCTGATTGGCGGTCATGACACGGCCTCTGTGGCATGTACCTTTCTTGTCAAGTACCTCGGAGAGCTGCCACATATCTACGACAAGGTCTACCAGGAGCAGATGGAGATCGCCAAGAGTAAACCGGCCGGCGAGCTGCTGAACTGGGATGATCTCAAGAAGATGAAGTATTCGTGGAACGTTGCATGCGAGGTCATGCGGCTCAGCCCTCCTCTCCAAGGTGGCTTCCGAGAGGCAATCACCGACTTTATGTTCAACGGCTTTTCAATTCCCAAGGGATGGAAGCTGTACTGGTCGGCCAACTCGACTCACAAGAACGCCGAGTGTTTCCCCATGCCTGAGAAGTTCGACCCCACACGCTTTGAGGGAAACGGCCCAGCTCCTTACACCTTTGTCCCTTTTGGTGGAGGTCCCCGAATGTGCCCCGGCAAGGAGTACGCCCGTCTCGAAATTCTGGTGTTCATGCACAACCTGGTCAAGAGATTCAAGTGGGAGAAGGTGATCCCGGATGAAAAAATCATTGTTGATCCGTTTCCCATTCCTGCCAAGGACCTGCCCATTCGACTCTACCCCCATAAAGCTTGA

***CaCYP716A83 (C. asiatica)* Sequence (5'-3')**

ATGGAGCTGTTTTTTGTGCCTCTGTTTAGCTCGCTTGTTTTGTTTGTGTTTTTCTGCTTCTTGCTCCTGTTCTACAAGAACAACAAGTGGAGATCCTCTGGTGCCCCGCTGCCTCCCGGCCAGACTGGCTGGCCCTTCATCGGAGAGAGCTACGAGTTCTTGTCTACCGGATGGAAGGGCTACCCGGAGAAGTTCATCTTCGATCGGATCGCCAAGCACTCTTCTAATGTGTTCAAGACATCAATCCTGGGTGAGCATGCTGCCGTCTTTTGTGGTGCTGCTTGCAACAAGTTCCTGTTCTCGAACGAGAATAAGCTGGTCCAGGCCTGGTGGCCTGACTCCGTCAACAAGGTGTTCCCTAGTAGTACCCAAACGTCATCCAAGGAGGAAGCCATTAAAATGCGAAAGATGCTGCCCAACTTCCTTAAGCCCGACGCTCTCCAGAGATATGTTGGAACCATGGACTCGATAGCGCGTAGACATTTTGAATCAGGCTGGGACAACAAGAATGAAATTGTCGTTTTCCCCCTAGCTAAGACATACACGTTCTGGATCGCTTGCAAGCTTTTTGTCAGTGTCGAGGAGCCCTCGCAGGTTGCTAAATTGCTGGAGCCCTTTTCGGCTATCGCCTCTGGAATTATTTCTGTTCCTATTGACCTGCCAGGCACCCCTTTCAACTCTGCGATTAAGTCCTCCAAGCGCATTCGCGAGATGCTGTGGAAGATAATTAAGCAGCGAAAAATTGATCTCGCAGAAGGTAAAGCCTCTCCCACTCAAGATATCCTATCCCACATGCTCTTGACTAGCGATGAGAACGGTAAATTCATGAGCGAACTTGATATTGCAGACAAGATTCTTGGGCTTCTCATTGGTGGCCACGACACAGCATCTTCTGCCTGTACTTTCGTGGTGAAATTTCTCGCCGAGCTCCCAGAAATCTACGACGGTGTGTATAAGGAACAGATGGAGATCGTCAAGAGCAAGGGACCTGGGGAACTGCTCAACTGGGATGACATCCAGAAGATGAAGTACTCGTGGAATGTCGCGTGCGAGGTGCTGCGACTGGCCCCACCTCTCCAGGGCGGCTTCCGAGAGGTTCTCACCGACTTCTCGTACAACGGATTTTCCATTCCCAAAGGCTGGAAGATCTACTGGACCGCCAACTCCACCCATCGAAACTCCGAGGTGTTCCCTGAGCCTCTCAAGTTTGACCCGTCACGGTTCGAGGGAACTGGGCCGCCCCCGTTCACCTTTGTACCCTTTGGAGGAGGACCCCGTATGTGTCCAGGCAAGGAGTACGCCCGACTTGAGATTCTGGTGTTCATCCACAACCTCGTGAAACGGTATAAATGGGAGAAGATCATCCCTGATGAAAAGATCATTGTTAACCCCATGCCCATCCCTGCAAAGGGTCTGCCTATTCGACTGTTTCCCCACAAGGCCTGA

***CrCYP716AL1 (C. roseus)* Sequence (5'-3')**

ATGGAAATCTTCTACGTTACTCTTTTGTCTCTGTTTGTCCTGCTGGTGTCTCTTAGCTTCCACTTTCTTTTCTACAAGAATAAATCGACGCTGCCTGGCCCATTACCACCCGGACGAACCGGCTGGCCCATGGTGGGTGAGTCCCTGCAATTTCTCTCTGCTGGATGGAAGGGCCACCCCGAGAAGTTTATCTTTGATCGAATGGCCAAGTATTCGTCTAATGTTTTCCGATCACATCTGCTCGGCGAACCCGCAGCCGTCTTCTGTGGTGCGATTGGCAACAAATTTCTTTTTAGCAACGAGAACAAGTTGGTCCAGGCCTGGTGGCCAGATAGCGTTAACAAGGTGTTCCCATCTAGTAACCAGACCTCATCGAAGGAGGAGGCCATCAAGATGCGTAAAATGCTTCCTAACTTCCTCAAGCCCGAGGCTCTCCAAAGATACATCGGTTTGATGGACCAGATTGCCCAAAAGCATTTCTCGTCTGGTTGGGAAAACCGAGAACAGGTGGAGGTGTTTCCTCTGGCAAAGAACTACACTTTCTGGCTGGCTTCCCGACTCTTTGTGTCTGTTGAAGATCCCATTGAGGTTGCCAAGCTCCTGGAACCGTTTAATGTGCTCGCGTCTGGTCTCATCTCTGTGCCCATAGATCTTCCCGGCACTCCTTTCAACAGAGCCATCAAGGCTTCTAATCAGGTTCGGAAAATGCTCATTTCCATCATTAAGCAGCGAAAAATTGACCTGGCCGAGGGCAAGGCATCGCCTACCCAGGACATTCTGTCCCACATGCTGTTGACCTCCGACGAGAATGGGAAATTCATGCATGAGCTGGATATAGCAGACAAGATTCTTGGACTTCTCATCGGCGGCCACGACACAGCGTCCTCCGCTTGCACATTCATTGTGAAGTTTCTGGGTGAGCTCCCGGAGATCTACGAGGGAGTCTATAAGGAGCAGATGGAGATTGCCAACTCCAAGGCGCCTGGAGAGTTCTTAAACTGGGAAGATATTCAGAAGATGAAGTACTCGTGGAACGTGGCCTGCGAGGTTCTGCGTCTCGCTCCTCCCTTGCAGGGCGCCTTTCGCGAAGCTCTAAACGACTTCATGTTCCATGGATTCAGTATTCCCAAGGGATGGAAGATTTACTGGAGCGTCAACTCTACGCACCGAAACCCTGAGTGCTTCCCCGACCCCCTCAAGTTCGACCCGAGTCGGTTTGACGGGTCGGGACCTGCTCCTTATACCTTCGTACCCTTTGGAGGTGGCCCTCGCATGTGTCCCGGAAAGGAGTACGCTCGGCTGGAGATTCTCGTCTTTATGCACAACCTGGTCAAGCGATTCAAGTGGGAGAAGATCATCCCCAACGAAAAGATCGTAGTCGACCCTATGCCGATCCCAGAGAAAGGTCTTCCTGTGAGACTATACCCCCACATCAACGCCTGA

***OeCYP716A48 (Olea europaea)* Sequence (5'-3')**

ATGGAGTTTTTCTACGTGAGTCTTCTGTGTCTGTTTGTGTTTTTGATCTCCCTGTCTTTGCATTTTCTCTTCTACAAGAACAAGTCTAGCTTTTCCGGACAAATACCGCCGGGCAAGACCGGCTGGCCCGTCATTGGCGAATCTCTGGAGTTCTTGAGCAATGGGTGGAAGGGCCATCCCGAAAAGTTCATTTTCGACCGAATCGCCAAATACTCGTCATACGTGTTCCGAACTCATCTTTTTGGAGAGCCAGCTGCCGTTTTCTGTGGAGCCAACGGCAATAAGTTTCTCTTTTCGAATGAGAACAAACTCGTACAGGCCTGGTGGCCTGCTTCCGTTGACAAGGTTTTCCCCTCCAGCAACCAGACTTCTTCTAAGGAGGAGGCGGTGAAGATGCGAAAAATGTTGCCGACTTTCTTCAAGCCCGAGGCTCTCCAACGGTACGTTGGAATAATGGACCACATTGCCCAGCGACACTTCTCCGACGGTTGGGACAACAAGAATGAGGTCGTGGTTTTCCCTCTAGCAAAGAGGTACACCTTCTGGCTGGCATGTCGACTTTTCGTCTCTGTGGAGGATCCCGCCCACGTCGCCAAGTTTGCCGACCCATTCAACGAGCTAGCATCTGGCCTTATTAGCATTCCTATTGATCTGCCTGGTACTCCCTTCCACCGAGCCATTAAATCGTCCAACTTCATCCGTAAGGAACTGGTGTCGATCATCAAGCAGCGCAAGATCGACCTGGCCGAGGGAAAGGCCTCACCCACCCAAGACATTCTGTCACACATGCTGCTGACATCGGACGAGAGTGGTAAATTCATGCATGAGCTGGATATCGCTGATAAAATCCTCGGTCTGTTAGTCGGAGGCCACGACACAGCTTCTTCCGCTTGCACCTTTGTCGTGAAATACCTGGCTGAACTTCCTGAAATTTACGAAGGAGTCTACCAGGAGCAGATGGAGATCGCGAAATCCAAGGCGCCCGGTGAGCTGCTCAACTGGGATGACATCCAGAAGATGAAGTATTCATGGAACGTGGCTTGCGAGGTTCTGCGGCTTGCGCCTCCTCTGCAGGGAGCTTTCAGAGAGGCCATCACGGACTTCATGTTCAACGGGTTTTCTATTCCCAAGGGTTGGAAGCTCTACTGGTCTGCTAACTCCACCCACAGAAACAGTGAATTCTTTCCTGAGCCACTCAAGTTCGATCCGTCAAGATTTGAGGGCTCGGGGCCTGCTCCCTATACGTTTGTCCCCTTTGGCGGAGGACCCCGAATGTGCCCGGGCAAGGAGTACGCCCGGCTCGAAATCCTCGTGTTTATGCACCATCTGGTCAAGCGATTCAAGTGGGAGAAGCTCATCCCTGATGAGAAGATTGTGGTTGACCCCATGCCCATTCCAGCCAAGGGTCTCCCTATTCGTCTATATCCCCTGAACGCATGA

**References**

[1] Ning Y, Liu M, Ru Z, Zeng W, Liu S, Zhou J. Efficient synthesis of squalene by cytoplasmic-peroxisomal engineering and regulating lipid metabolism in Yarrowia lipolytica. Bioresour Technol 2024;395:130379. https://doi.org/https://doi.org/10.1016/j.biortech.2024.130379.
